# Microplastics as evolving particle–chemical–microbial interfaces: plastisphere ecology, ecotoxicity, and safety-informed remediation

**DOI:** 10.3389/fmicb.2026.1875310

**Published:** 2026-10-13

**Authors:** Haixin Jiao, Ahmed E. M. Fodah, Michael Schagerl, Bairen Yang, Sameh S. Ali

**Affiliations:** 1 School of Environmental Science and Engineering, Yancheng Institute of Technology, Yancheng, China; 2 Biofuels Institute, School of the Environment and Safety Engineering, Jiangsu University, Zhenjiang, China; 3 State Key Laboratory of Coal Combustion, School of Energy and Power Engineering, Huazhong University of Science and Technology, Wuhan, China; 4 College of Agricultural Engineering, Al-Azhar University, Cairo, Egypt; 5 Department of Functional and Evolutionary Ecology, University of Vienna, Vienna, Austria; 6 Botany and Microbiology Department, Faculty of Science, Tanta University, Tanta, Egypt

**Keywords:** antimicrobial resistance, biogeochemical cycling, ecotoxicology, environmental remediation, microbial ecology, microbial transformation, microplastics, plastisphere

## Abstract

Microplastics (MPs) are not environmentally static particles; aging, fragmentation, contaminant acquisition, organic conditioning, and microbial colonization progressively convert them into dynamic particle–chemical–microbial interfaces. This transformation is central to their environmental fate and risk because plastisphere communities can alter particle transport, contaminant partitioning, nutrient cycling, antimicrobial-resistance dynamics, and polymer transformation. Experimental evidence shows that MP biofilms can increase conjugative transfer of antibiotic-resistance genes by approximately 7.2–19.6-fold relative to suspended bacteria, illustrating that microbial colonization can modify ecological function rather than merely change community composition. However, microbial presence, functional-gene enrichment, surface deterioration, and polymer mass loss should not be interpreted as equivalent evidence of biodegradation. Stable-isotope studies show that even biodegradable polymers may mineralize slowly and incompletely; approximately 65% mineralization of ^1^^3^C-labeled PBS required 425 days, whereas direct mineralization of persistent polyolefins remains far more limited. Ecotoxicological effects are similarly conditional on particle size, morphology, aging, associated chemicals, exposure duration, and environmental context, and evidence for trophic transfer is stronger than that for generalized biomagnification. Interpretation is further constrained by substantial analytical heterogeneity in sampling, size cut-offs, recovery, contamination control, polymer identification, and number-vs.-mass reporting, making methodological harmonization and uncertainty assessment essential for cross-study comparison. Remediation should therefore distinguish particle capture from destruction and biodeterioration from depolymerization, microbial assimilation, mineralization, and true upcycling. Although enzymatic and microbial conversion technologies show considerable promise, most high-performance demonstrations involve defined polymers, post-consumer plastics, or prepared hydrolysates rather than heterogeneous environmental MPs. Safe application consequently requires carbon and contaminant mass balances, residue and product safety testing, microbial containment, and life-cycle verification. Climate-sensitive transport and toxicity further reinforce the dynamic nature of MP exposure, while circular-economy strategies should prioritize prevention, low-shedding design, reuse, and recovery before attempting energy-intensive reclamation of dispersed environmental particles. Overall, a microbiology-informed assessment of MPs requires progression from microbial identity to demonstrated function, from particle detection to exposure relevance, and from apparent removal or degradation to verified net risk reduction.

## 1 Introduction

Microplastics (MPs), generally defined as synthetic polymer particles smaller than 5 mm, are now widely documented across marine, freshwater, terrestrial, and atmospheric environments. Recent global-scale analyses further demonstrate that their occurrence is both widespread and highly heterogeneous. Chen et al. (2026) compiled 6,464 georeferenced water-sample concentration records from rivers, lakes, and oceans collected between 2014 and 2024 and identified pronounced spatial differences among aquatic systems, together with substantial disparities in monitoring coverage and reporting practices. In marine surface waters, Liu C. et al. (2026) and Liu X. et al. (2026) integrated observations from the upper 0–5 m collected between 1998 and 2023 and estimated a global burden of approximately 3.29 × 10^1^^4^ MP items, predominantly within the 0.33–5 mm size range. These large-scale assessments confirm that MP pollution is not restricted to isolated hotspots and also emphasize that apparent global patterns remain sensitive to particle size, sampling strategy, environmental compartment, and analytical methodology. Environmental exposure therefore cannot be interpreted from concentration alone because ultraviolet irradiation, oxidation, mechanical abrasion, aggregation, interaction with natural organic matter, and microbial colonization progressively modify particle size, surface chemistry, density, reactivity, and mobility (Xu et al., 2024; Zhao et al., 2025). Consequently, environmental exposure cannot be interpreted solely from the abundance or polymer identity of pristine particles; MPs acquire new physicochemical and biological properties as they move through interconnected ecosystems.

A particularly important component of this environmental transformation is microbial colonization. Plastic surfaces provide persistent substrates for bacteria, archaea, fungi, microalgae, and other microorganisms, producing structured biofilm communities collectively termed the “plastisphere” (Ormsby and Quilliam, 2026). These communities can differ from microorganisms in the surrounding water or natural particulate substrates and can modify surface charge, hydrophobicity, aggregation, effective density, and particle settling through production of extracellular polymeric substances and accumulation of biomass. Plastisphere activity can also influence nutrient cycling, contaminant transformation, and microbial dispersal; for example, experimental exposure of salt-marsh sediments to different polymer types altered microbial community composition and produced polymer-dependent effects on nitrification and denitrification (Wijesooriya et al., 2023). The microbiological significance extends to the environmental resistome: an experimental estuarine study reported that biofilms developed on MPs increased conjugative transfer frequencies of antibiotic-resistance genes by approximately 7.2–19.6-fold relative to suspended bacteria, with polyethylene-associated biofilms producing the strongest enhancement among the tested polymers (Zhou et al., 2024). These observations demonstrate that microbial colonization is not merely a surface phenomenon but can modify ecological functions and microbial interactions relevant to environmental and public-health risk.

Microbial processes operate within a broader network of physicochemical transformations that determines the ecotoxicological behavior of MPs. Weathering increases surface roughness and oxidation, promotes fragmentation toward the nanoscale, and can modify the sorption and subsequent release of metals and hydrophobic organic contaminants (Romarate et al., 2026). In one controlled aging study, cadmium adsorption onto polyethylene particles increased by approximately 40% following 30 days of aging, illustrating how environmental transformation can change contaminant-carrying capacity (Zhang et al., 2024). Biofilm formation adds another layer of complexity because extracellular polymeric substances and microbial metabolism can modify contaminant partitioning, speciation, and transformation. These processes may reduce the bioavailability of some compounds while facilitating the persistence, transport, or generation of transformation products from others (Bhagwat et al., 2021; Amaral-Zettler et al., 2020). MPs should therefore be considered evolving particle–chemical–microbial interfaces rather than inert polymer fragments, with hazard profiles determined jointly by polymer properties, environmental aging, associated contaminants, microbial community structure and activity, and biological exposure.

This dynamic environmental identity is directly relevant to ecotoxicity. MPs can exert physical effects through ingestion, abrasion, obstruction, and tissue translocation, while additives and sorbed pollutants can generate chemical co-exposures and contribute to oxidative stress, inflammation, endocrine disruption, genotoxicity, and other biological responses. Fragmentation into smaller micro- and nanoplastic particles can increase biological accessibility, whereas trophic transfer and redistribution among environmental compartments extend exposure beyond the site of initial release (Weis and Alava, 2023; Ma et al., 2026). At the same time, microbial activity can modify these pathways by changing particle buoyancy and aggregation, transforming associated chemicals, affecting biogeochemical cycling, and potentially facilitating the dispersal of opportunistic pathogens or resistance determinants (Balta et al., 2025). The environmental hazard of MPs is therefore not static: physical, chemical, and microbiological transformations continuously reshape both exposure and toxicity.

The same complexity must be considered when moving from pollution assessment toward remediation and circular-economy strategies. Resource-recovery approaches increasingly seek to transform plastic waste into recovered polymers, monomers, fuels, carbonaceous materials, or biologically derived products rather than treating plastics exclusively as wastes (Nasser et al., 2025; Wu, 2026). Environmental MPs, however, differ fundamentally from virgin polymers and many conventional post-consumer plastic feedstocks because they are generally heterogeneous in polymer composition and size, environmentally aged, biofouled, dispersed within mineral or organic matrices, and potentially associated with metals, persistent organic contaminants, additives, microorganisms, and degradation products (Srivastava et al., 2025). Consequently, conversion efficiencies demonstrated using clean polymers, standardized model MPs, sorted post-consumer plastics, or bulk plastic-waste streams cannot automatically be considered evidence of successful valorization of recovered environmental MPs. This distinction is particularly important for biological conversion, for which surface colonization, partial depolymerization, assimilation of released monomers, and complete mineralization represent different processes and should not be treated as equivalent outcomes (Jiao et al., 2026). Similarly, chemical and thermochemical conversion can redistribute contaminants into gases, liquids, chars, or process residues if feed characterization, decontamination, emission control, and product qualification are inadequate (Afifah et al., 2024). A credible circular strategy must therefore demonstrate not only conversion or product yield but also net risk reduction.

A further challenge is that the available evidence is methodologically heterogeneous. Reported MP concentrations and biological effects vary with sampling design, lower particle-size cut-offs, contamination control, extraction and recovery procedures, polymer-identification methods, particle morphology, environmental aging, exposure concentration, and reporting units. The recent synthesis of 1,885 ocean stations, for example, identified the lack of methodological standardization as a major source of uncertainty in estimating global MP distributions (Zhao et al., 2025). Comparable limitations affect ecotoxicological and microbial studies, in which pristine spherical particles at relatively high experimental concentrations are often used to infer effects of irregular, aged, biofilm-coated environmental particles (You et al., 2026). Evidence supporting valorization is similarly heterogeneous, ranging from controlled experiments on pure polymers to post-consumer waste and, much less frequently, recovered environmental MPs. Maintaining these distinctions is essential for separating demonstrated evidence from plausible extrapolation and for identifying where apparent inconsistencies among studies reflect differences in analytical or experimental design rather than genuine biological disagreement.

Several recent reviews have made important contributions within individual parts of this rapidly expanding field. Liu and Zheng (2025) synthesized global MP migration, transformation, degradation, ecological impacts, and human-exposure risks, whereas Liu et al. (2025) focused on aquatic systems and emphasized effects on water quality, biogeochemical cycles, microbial functions, pathogen dispersion, and emerging contaminants. Ansari et al. (2025) reviewed biological impacts, ecotoxicity, and human-health risks, while Balta et al. (2025) examined the more specialized interaction among MPs, heavy metals, microbial communities, and antimicrobial resistance. In parallel, Jiao et al. (2026) specifically addressed microbial and enzymatic MP biodegradation, distinguishing surface modification, depolymerization, biotransformation, and mineralization and examining engineered microbial systems and biosafety. Collectively, these reviews demonstrate major progress but also reveal that environmental fate, plastisphere ecology, mechanistic ecotoxicology, biodegradation, and plastic-waste conversion are still commonly evaluated as separate research domains.

The remaining need is a critical synthesis of the interfaces among these domains, particularly how microbial–environment interactions modify particle fate and hazard and how this mechanistic knowledge can define safety requirements for subsequent management and valorization. Therefore, the present review examines MP pollution through an integrated exposure–transformation–risk–management continuum, with microbial processes serving as a cross-cutting mechanistic component rather than an isolated topic. Specifically, the review aims to: (i) establish the environmental sources, characteristics, and exposure pathways necessary to interpret microbial colonization and biological contact; (ii) evaluate physical, chemical, biological, and microbially mediated mechanisms of ecotoxicity; (iii) determine how aging, natural organic matter, plastisphere formation, contaminant interactions, and fragmentation modify environmental fate and hazard; (iv) assess longer-term ecological and human-exposure implications while explicitly recognizing methodological and analytical uncertainties; and (v) critically evaluate mechanical, chemical, thermochemical, and biological valorization routes according to both technological performance and their capacity to avoid transferring contaminants or toxicity into products, emissions, or residues. Particular emphasis is placed on plastisphere ecology, biofilm-mediated transformation, microbial effects on biogeochemical processes and contaminant fate, and contained microbial or enzymatic conversion because these processes directly connect environmental microbiology with ecotoxicology, remediation, and risk-informed circularity. By integrating these dimensions and explicitly distinguishing direct evidence from environmental MPs from extrapolation based on model particles or broader plastic-waste streams, this review seeks to provide a microbiology-informed framework for determining not only how MP risks evolve in the environment, but also which interventions can credibly reduce those risks.

## 2 Bibliometric evidence and thematic evolution of microplastic research

The rapid expansion of MP research has created an evidence base that is increasingly difficult to interpret through conventional descriptive review alone. Bibliometric analyses consistently show that the field has moved from a relatively small literature focused primarily on occurrence and distribution toward a multidisciplinary research domain spanning environmental chemistry, ecotoxicology, microbiology, analytical science, human exposure, remediation, and resource recovery. In aquatic systems, Yang et al. (2025) identified 1,141 original articles published between 2013 and 2023, with annual output increasing from only two articles in 2013 to 289 in 2023. Notably, 1,025 of these publications−89.8% of the total dataset—appeared during the final 5 years of the study period. The disciplinary breadth expanded simultaneously: the number of Web of Science research categories represented in this literature increased from three in 2013 to 35 by 2023. Marine environments dominated the evidence base, but rivers, wastewater, and lakes became increasingly prominent research systems (Yang et al., 2025). This expansion has not been confined to aquatic pollution. Bibliometric analysis of plastic pollution and soil microbial communities identified 452 publications in the Web of Science Core Collection and demonstrated a pronounced acceleration after 2018, with annual publication output reaching 193 articles in 2023 (Yang et al., 2024). The transition is important because it marks a shift from treating MPs primarily as physical contaminants toward examining their interactions with microbial communities, soil physicochemical properties, nutrient cycling, ecological functions, and co-occurring pollutants. MP research has therefore become progressively less compartmentalized by environmental medium and increasingly concerned with mechanisms operating across environmental and biological interfaces. The rapid growth of the literature also explains why another broad catalog of MP sources, occurrence, and generic toxicity would have limited added value. Bibliometric evidence instead indicates that the scientific frontier now lies in understanding the relationships among processes that were previously studied separately.

### 2.1 Keyword co-occurrence and microbiological thematic evolution in microplastic research

Bibliometric patterns increasingly position microorganisms as integral components of MP fate and risk rather than as a specialized subtopic. In the aquatic literature, “microbial community” emerged among the prominent recent keywords by 2022, alongside ecological risk and risk assessment (Yang et al., 2025). This transition parallels the rapid development of plastisphere research, in which plastic surfaces are considered distinct microbial habitats capable of influencing particle transport, contaminant interactions, nutrient transformations, pathogen ecology, and antimicrobial-resistance dynamics. The trend is particularly clear in terrestrial systems. Keyword co-occurrence analysis by Yang et al. (2024) identified major research themes involving soil bacterial communities, microbial diversity, nitrogen cycling, ecosystem functioning, heavy metals, plastisphere ecology, biodegradable plastics, ecotoxicity, fungi, nanoplastics, analytical methods, and human health. Time-resolved clustering further identified “microbial community,” “analytical methods,” “microbial diversity,” “nutrient cycling,” “degradable microplastics,” “emerging contaminants,” and “human health” as interconnected research trajectories. Importantly, recent thematic development has shifted toward mechanisms linking plastic residues with microbial functions, combined pollution, soil enzymatic activity, plastisphere and rhizosphere diversity, food-web effects, and ecosystem health (Yang et al., 2024). These patterns reinforce the concept developed in this review that MP risk cannot be separated from the microbial and biogeochemical context in which particles age and accumulate. A parallel bibliometric trajectory is evident in biodegradation research. Cao et al. (2024) screened 545 records and retained 331 studies addressing MP biodegradation and associated mechanisms, with particularly rapid growth during 2018–2022. The dominant topics included screening of functional microorganisms, identification of plastic-active enzymes, and elucidation of degradation mechanisms. Reported functional groups were concentrated among bacterial and fungal taxa, particularly Proteobacteria, Firmicutes, and Ascomycota, while hydrolases, oxidative enzymes, and depolymerases represented major enzyme classes investigated in the literature (Cao et al., 2024). This development reflects a shift from descriptive observations of microbial colonization toward functional questions involving depolymerization, metabolic utilization, and engineered biological transformation. However, growth in publication volume should not be interpreted as equivalent to maturation of the evidence. Colonization, visible surface deterioration, polymer-chain cleavage, oligomer or monomer release, microbial assimilation, and mineralization represent biologically distinct outcomes. Bibliometric expansion in biodegradation research has occurred faster than the development of standardized criteria for demonstrating these different stages. This distinction is particularly important when biodegradation findings are subsequently invoked to support environmental remediation or circular-biotechnology applications.

### 2.2 Convergence toward particle–chemical–microbial interactions

The thematic expansion of the field also demonstrates increasing convergence between MP research and conventional contaminant science. Early studies emphasized particle abundance, distribution, morphology, and polymer type, whereas more recent work increasingly treats MPs as reactive interfaces capable of interacting with metals, persistent organic contaminants, pharmaceuticals, antibiotics, dissolved organic matter, and microorganisms (Kinigopoulou et al., 2022; Shaji et al., 2025; Mousazadehgavan, 2026). In the soil-microorganism literature, heavy metals appear among the highly connected keywords, while emerging-contaminant clusters increasingly intersect with microbial diversity, soil health, and ecological-risk research (Yang et al., 2024). Similarly, bibliometric analysis of MP–heavy-metal research has identified biofilms as central components linking physicochemical sorption with microbial processes and combined toxicity. This shift has important implications for interpretation of ecotoxicological evidence. MPs entering natural environments are progressively altered by oxidation, mechanical weathering, natural organic matter, microbial extracellular polymers, biofilm development, and contaminant sorption. These processes modify particle surface properties and biological interactions, meaning that ecotoxicological outcomes obtained with pristine particles may not directly predict responses to environmentally aged and colonized particles. The bibliometric transition toward terms such as ecological risk, microbial community, biodegradation, heavy metals, and human health therefore reflects more than the emergence of new topics; it represents recognition that MP exposure is a coupled particle–chemical–microbial problem. Recent work focused specifically on marine MP–microorganism interactions reinforces this convergence. Wang et al. (2026) integrated bibliometric mapping from 2015 to 2025 with mechanistic synthesis and identified plastisphere development, microbial community restructuring, pollutant and antimicrobial-resistance transport, biofilm-mediated aggregation, enzymatic degradation, and biogeochemical cycling as central emerging themes. Their analysis further emphasized a persistent gap between molecular- or community-level microbial observations and ecosystem-scale consequences, particularly in sedimentary and deep-ocean environments. Thus, although the microbiological dimension of MP pollution has expanded rapidly, mechanistic links across scales remain incompletely resolved.

### 2.3 Transition from pollution characterization toward remediation and resource recovery

A second major bibliometric transition is the increasing emphasis on intervention. Analysis of 1,892 Scopus-indexed publications on MP pollution and removal in water between 2011 and 2024 showed strong publication growth, with 557 publications appearing in 2024 alone (Mai et al., 2025). Keyword analysis identified five major thematic areas: wastewater treatment, MP degradation, pollution risk, advanced removal technologies, and adsorption-based treatment. Emerging research directions included biochar adsorption, electrocoagulation, membrane filtration, and nature-based treatment approaches such as constructed wetlands (Mai et al., 2025). Collectively, these trends demonstrate that MP science is moving beyond occurrence and hazard identification toward removal and treatment. Nevertheless, a conceptual gap remains between removal, degradation, and safe valorization. Physical removal transfers MPs from one environmental compartment into a concentrated waste stream; degradation may generate smaller particles, oligomers, dissolved compounds, or metabolites; and valorization seeks to transform recovered polymer carbon into useful products. These are not equivalent endpoints. A technology may achieve high particle-removal efficiency without eliminating the associated chemical or biological hazard, while a conversion process may achieve high polymer yield but redistribute contaminants into gaseous emissions, liquid products, char, process water, or residues. The bibliometric structure of the field reflects this separation. Removal studies are dominated by environmental engineering and water-treatment themes, biodegradation studies by microbial and enzymatic mechanisms, and plastic conversion studies largely by chemical and process engineering. Ecotoxicological validation, contaminant fate, product safety, microbial containment, and life-cycle consequences are much less consistently integrated into the same analytical framework. This fragmentation becomes particularly important when technologies demonstrated on virgin polymers, intentionally prepared MPs, or sorted post-consumer plastic waste are extrapolated to environmental MPs that are aged, compositionally heterogeneous, and burdened with co-contaminants.

### 2.4 Analytical uncertainty as an emerging cross-cutting limitation

Bibliometric development has also highlighted an increasingly important methodological problem: rapid growth in publication volume has not been accompanied by equivalent harmonization of analytical methods. Analytical methodology appears as a distinct research cluster in microbiome-focused terrestrial MP research (Yang et al., 2024), while recent bibliometric assessments of aquatic pollution and treatment identify standardization of detection and characterization as a continuing research priority (Yang et al., 2025; Mai et al., 2025). This limitation affects nearly every major evidence domain considered in this review. Environmental abundance estimates depend on sampling strategy, mesh or filter size, digestion and extraction procedures, recovery efficiency, contamination control, polymer-identification method, and reporting units. Microbial studies additionally differ in biofilm incubation time, polymer aging, environmental matrix, sequencing or cultivation approach, and whether taxonomic profiles are accompanied by functional measurements. Ecotoxicological experiments vary in particle size, shape, concentration, polymer type, surface state, exposure duration, and biological model. Consequently, apparently conflicting results may arise not from true biological inconsistency but from methodological non-equivalence. The same issue influences human-exposure studies, where microscopy, μFTIR, Raman spectroscopy, pyrolysis-GC/MS, and related techniques differ fundamentally in particle-size resolution, chemical specificity, mass-vs.-particle-number reporting, sample destruction, and susceptibility to contamination or spectral interference. It also affects valorization studies because conversion performance obtained using homogeneous polymers under controlled conditions is frequently compared with results from mixed or contaminated feeds. Analytical uncertainty should therefore be regarded not as an isolated technical issue, but as a cross-cutting determinant of evidence quality throughout MP science.

### 2.5 Knowledge fragmentation and positioning of the present review

Taken together, published bibliometric analyses reveal a field that has expanded rapidly but remains organized into partially disconnected research domains. Aquatic studies have evolved from occurrence toward risk assessment and management; soil research increasingly emphasizes microbiome structure, nutrient cycling, ecological risk, and combined pollution; biodegradation research concentrates on functional microorganisms and enzymes; and treatment research is increasingly focused on engineered removal technologies (Cao et al., 2024; Yang et al., 2024, 2025; Mai et al., 2025). More specialized recent syntheses have begun to integrate plastisphere ecology with marine biogeochemical cycling and microbial transformation, but the connection between these environmental processes and downstream safety criteria for remediation and resource recovery remains incompletely developed (Wang et al., 2026). This fragmentation defines the rationale for the present review. Rather than reproducing the established literature on MP occurrence or treating microbial ecology, ecotoxicity, environmental transformation, and valorization as parallel topics, we organize them as sequentially connected components of an exposure–transformation–risk–management continuum. The central premise is that environmental transformation—including aging, contaminant sorption, natural-organic-matter interactions, and plastisphere development—changes the physical, chemical, and biological identity of MPs; that this evolving identity determines exposure and hazard; and that these characteristics must subsequently define the requirements for safe removal, degradation, and valorization. Accordingly, the following sections move from environmental exposure and transformation toward plastisphere ecology and microbially mediated contaminant processes, then to ecotoxicological consequences and analytical uncertainty, before evaluating degradation, remediation, and valorization according to evidence directness and safety. This structure deliberately distinguishes environmental MPs from pristine model particles and conventional plastic-waste feedstocks and separates demonstrated environmental performance from mechanistic or technological extrapolation. In doing so, the review uses the bibliometric evolution of the field not simply to identify popular topics, but to expose the interfaces where evidence remains fragmented and where microbiology-informed synthesis is most needed.

## 3 Environmental exposure and transformation

### 3.1 Source-related particle properties define the initial state of environmental exposure

The traditional primary–secondary classification remains useful for identifying origins but becomes less informative when it obscures differences in particle formation and composition. For the purposes of environmental fate, three operational categories are more informative: intentionally manufactured microscopic particles, particles generated during product use, and particles produced through environmental fragmentation of larger plastics. Tire-road-wear particles illustrate the importance of this distinction. Across the countries evaluated by Kole et al. (2017), estimated tire-wear emissions ranged from approximately 0.23-4.7 kg capita^−1^ year^−1^, with a global average of about 0.81 kg capita^−1^ year^−1^. These particles are composite materials containing elastomers, fillers, carbon black, metals, vulcanization products, and other additives; consequently, their environmental behavior cannot be adequately represented by data obtained from pristine polyethylene or polystyrene microspheres. Textile emissions demonstrate an analogous interaction between source and morphology. Browne et al. (2011) reported that laundering a single garment could release more than 1,900 fibers per wash, establishing domestic washing as an important pathway of fibrous plastic material into wastewater. The significance of this result extends beyond emission quantity because fibers possess high aspect ratios and hydrodynamic properties fundamentally different from spherical particles. Thus, source-related particle generation determines not only how many particles enter an environmental compartment but also the morphology, surface area, additive composition, and transport characteristics of the emitted material. Environmental fragmentation produces an even more heterogeneous source state. In North Atlantic samples, ter Halle et al. (2016) observed a change in particle morphology around the millimeter scale: larger particles were relatively flat and frequently displayed asymmetric photodegradation and biofilm coverage, whereas smaller fragments tended to be more compact and more uniformly weathered. Their analysis also identified a deficit of particles below approximately 1 mg, suggesting that fragmentation behavior changes as particle geometry evolves. The result challenges a simple cascade model in which large plastics progressively fragment into geometrically similar smaller particles. Instead, fragmentation modifies morphology and surface exposure, which subsequently changes susceptibility to radiation, abrasion, sorption, and biological colonization. Source information should therefore be retained in risk assessment only when it is connected to measurable particle properties.

### 3.2 Cross-compartment transport, retention, and remobilization

Environmental distribution is not a static map of contamination but the outcome of repeated transport, retention, and remobilization. Particle morphology can substantially modify these processes. Laboratory measurements by Tatsii et al. (2024) showed that gravitational settling velocities of airborne MP fibers were up to 76% lower than those of volume-equivalent spheres. When the experimentally constrained settling velocities were incorporated into atmospheric transport simulations, fibers approximately 1 mm in length exhibited a substantially greater probability of reaching remote regions, including the high Arctic, than equivalent spherical particles. These results demonstrate that treating irregular MPs as spheres can systematically underestimate atmospheric residence time and long-distance dispersal. Atmospheric measurements confirm that this pathway can generate appreciable environmental fluxes, but they also reveal an important analytical limitation. Dris et al. (2016) measured fiber deposition ranging from 2 to 355 particles m^−^^2^ day^−1^ during year-long monitoring at an urban site, with an average of 110 ± 96 particles m^−2^ day^−1^ compared with 53 ± 38 particles m^−^^2^ day^−1^ at a less urbanized site. Chemical characterization, however, indicated that only approximately 29% of the collected fibers were entirely synthetic or mixtures containing synthetic material. Consequently, microscopy-based fiber counts cannot automatically be interpreted as plastic counts, and failure to verify polymer identity may inflate estimates of atmospheric MP abundance.

Vertical transport in aquatic systems is equally dynamic. The biofouling model developed by Kooi et al. (2017) predicted that particles may remain afloat, sink, or undergo repeated vertical oscillations depending on particle size, polymer density, biofilm growth, light, and water-column properties. Under an 8-h-light/16-h-dark scenario, modeled settling was delayed for more than 116 days, whereas longer light exposure accelerated algal growth and reduced the onset time for sinking. Biofouling therefore does not simply convert floating particles into permanently sinking particles; it changes their temporal and vertical exposure distribution. This distinction is important because redistribution from surface waters toward intermediate or deeper layers changes the biological communities exposed without necessarily removing the particle from the ecosystem. Terrestrial transport provides further evidence that physical mobility cannot be inferred from particle density and pore-water flow alone. In a 21-day experiment, Rillig et al. (2017) applied polyethylene particles belonging to four size classes (710–850, 1,180–1,400, 1,700–2,000, and 2,360–2,800 μm) to soil surfaces. Without *Lumbricus terrestris*, particles remained predominantly in the upper soil layer; in the presence of earthworms, all size classes were redistributed downward to approximately 10 cm, with the smallest particles transported most effectively to the deepest layer. The interactive effects of earthworm presence, particle size, and soil depth were statistically significant (*P* < 0.001), demonstrating that bioturbation can create transport pathways not predicted by passive physical models.

These results collectively indicate that atmospheric deposition, hydrodynamic transport, sedimentation, biofouling, bioturbation, runoff, and resuspension operate as interconnected processes rather than independent endpoints. An environmental compartment may therefore function as a sink at one time and a secondary source at another. Persistence consequently involves both resistance of the polymer to molecular degradation and continued cycling of particulate plastic through environmental reservoirs.

### 3.3 Abiotic aging and fragmentation modify particle identity without necessarily eliminating polymer mass

Photo-oxidation converts relatively hydrophobic polymer surfaces into chemically heterogeneous interfaces through radical formation, chain scission, oxygen-containing functional groups, cracking, and embrittlement. The environmental consequence is not necessarily detoxification or disappearance. In accelerated sunlight-weathering experiments, Al Harraq et al. (2022) found that only 10 days of aging markedly increased MP dispersibility and resulted in more than a two-fold increase in surface uptake of model pollutants, including malachite green and lead ions. Partial polymer deterioration can therefore increase interfacial reactivity before appreciable mineralization occurs. Fragmentation provides an even clearer distinction between transformation and removal. Lambert and Wagner (2016) examined degradation-associated particle generation from several polymers over size ranges extending from approximately 30 nm to 60 μm. After 112 days, polystyrene generated approximately 92,465 particles mL^−1^ in the 2–60 μm fraction compared with 2,147 particles mL^−1^ in the control. In the 30–2,000 nm fraction, polystyrene reached approximately 6.4 × 10^8^ particles mL^−1^ after 84 days compared with approximately 0.2 × 10^8^ particles mL^−1^ in controls. These laboratory rates should not be extrapolated directly to natural environments, but they demonstrate that apparent degradation can substantially increase particle number while redistributing polymer mass toward smaller size fractions. This distinction is particularly important at the MP–nanoplastic transition. Increasing fragmentation raises the number of particle interfaces and surface-area-to-mass ratio while simultaneously moving material into size ranges that are difficult to quantify using conventional MP methods. Consequently, disappearance of visible particles, mass loss, cracking, or surface erosion cannot be interpreted as complete degradation unless smaller fragments, dissolved products, and ultimately mineralization are also evaluated. This principle becomes especially important when biological degradation is considered later in the manuscript.The interconnected transport, redistribution, weathering, and fragmentation pathways that determine the environmentally encountered particle are summarized in Figure 1.

**Figure 1:**
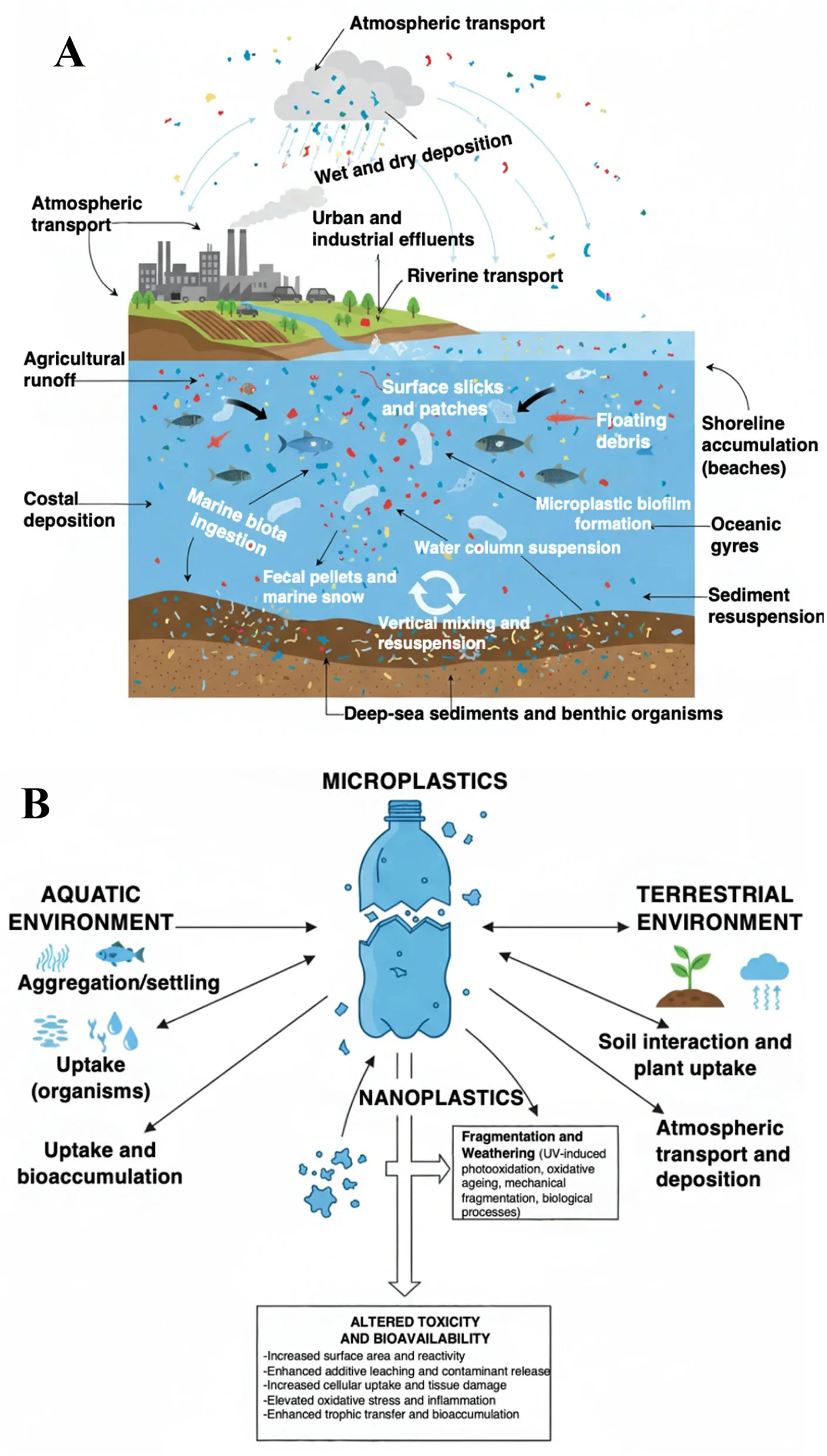
Environmental transport and transformation pathways of microplastics. **(A)** Cross-compartment transport and redistribution of microplastics through atmospheric transport and deposition, terrestrial and riverine inputs, surface-water transport, biological ingestion, marine-snow formation, vertical mixing, sedimentation, resuspension, shoreline accumulation, and benthic retention. **(B)** Transformation of microplastics across aquatic and terrestrial environments through aggregation, biological interactions, atmospheric redistribution, weathering, and fragmentation toward nanoplastics, with consequent changes in particle bioavailability and toxicity.

Aging additionally modifies interactions with co-occurring contaminants. Zhang et al. (2024) reported that after 30 days of aging, cadmium adsorption increased by 40.61% for polyethylene and 25.49% for polylactic acid, while chromium adsorption increased by 37.50 and 69.29%, respectively. Two-dimensional Fourier-transform infrared correlation spectroscopy indicated carbon-chain breakdown and the formation of oxygen-containing functional groups, whereas adsorption was associated with pore filling, electrostatic interactions, complexation, and hydrogen bonding. The strongly polymer- and metal-dependent response demonstrates that aging does not produce a uniform increase in contaminant affinity; rather, it generates new surface chemistries whose consequences depend on the interacting pollutant. Environmental aging rates nevertheless remain difficult to derive from accelerated experiments. Solar spectrum, temperature, oxygen availability, sediment burial, mechanical abrasion, organic coatings, and biological fouling differ considerably among environmental compartments. Jansen et al. (2024) concluded that solar ultraviolet radiation contributes to polymer deterioration, chemical release, and micro- and nanoplastic generation, but emphasized that these processes interact with temperature and other environmental drivers. Controlled weathering studies are therefore most useful for elucidating mechanisms, whereas environmental timescales require validation under realistic exposure histories.

### 3.4 Organic conditioning and contaminant acquisition create chemically dynamic interfaces

Interaction with natural organic matter begins soon after MPs enter waters, soils, sediments, or pore waters. Humic substances and other dissolved organic constituents can alter surface charge, steric interactions, aggregation, retention, and transport. In saturated porous-media experiments, Zhao et al. (2022) showed that humic acid increased the mobility of both 1.0- and 0.2-μm polystyrene particles, whereas increasing ionic strength from 1 to 100 mM potassium chloride reduced transport. The smaller 0.2-μm particles were more sensitive to both humic acid and ionic-strength changes, indicating that matrix effects become increasingly important as particle dimensions decrease. Thus, mobility cannot be predicted from polymer identity independently of surrounding water chemistry.

The same environmental conditioning affects chemical acquisition. Long-term field exposure of five common polymers by Rochman et al. (2013a) demonstrated progressive and polymer-dependent accumulation of polycyclic aromatic hydrocarbons (PAHs) and polychlorinated biphenyls (PCBs) during deployment in San Diego Bay. This establishes that environmental plastics can acquire persistent organic contaminants under realistic conditions. However, detection of a contaminant on a plastic surface does not establish that plastic is the dominant exposure vector because contaminant transfer to organisms additionally depends on fugacity gradients, desorption kinetics, ingestion rate, alternative sorbents, sediment exposure, and diet. This distinction was quantified by Koelmans et al. (2016), whose multimedia modeling estimated that approximately 98.3% of hydrophobic organic contaminants in the modeled ocean system resided in water, whereas only approximately 0.0002% was associated with plastic. Even after restricting the comparison to relevant solid phases, only about 0.02% of the contaminant mass was estimated to be plastic-associated. Their bioaccumulation modeling further indicated that natural prey and other environmental pathways often dominate chemical exposure. Accordingly, strong sorption observed under laboratory conditions should not automatically be interpreted as a major contribution to organism-level contaminant dose.

Antibiotics illustrate how polymer chemistry can nevertheless generate strong compound-specific associations. Li et al. (2018) compared five antibiotics across polyethylene, polystyrene, polypropylene, polyamide, and polyvinyl chloride and found that polyamide exhibited the greatest adsorption, with freshwater distribution coefficients ranging from 7.36 ± 0.257 to 756 ± 48.0 L kg^−1^. For polystyrene, polyethylene, polypropylene, and polyvinyl chloride, adsorption generally followed the sequence ciprofloxacin > amoxicillin > trimethoprim > sulfadiazine > tetracycline. Adsorption was substantially weaker in seawater, and no measurable adsorption of ciprofloxacin or amoxicillin was observed under the tested seawater conditions. These results demonstrate that statements describing MPs generically as antibiotic vectors conceal large differences arising from polymer functionality and aqueous chemistry. Natural organic matter can further suppress apparent carrier capacity. Atugoda et al. (2020) found that increasing ionic strength to 0.1 M sodium nitrate reduced ciprofloxacin adsorption to polyethylene by approximately 17%, whereas addition of humic acid reduced adsorption by approximately 89%. Ciprofloxacin adsorption was also strongly pH dependent, reaching a maximum around pH 6.5–7.5. The magnitude of the humic-acid effect demonstrates that adsorption measured using simplified laboratory water can substantially overestimate contaminant association under organic-matter-rich environmental conditions. Taken together, these findings support a conditional rather than universal interpretation of MPs as contaminant vectors. Metals, antibiotics, persistent organic pollutants (POPs), and other chemicals may associate strongly with particular polymers or aged surfaces, yet their environmental significance depends on competitive sorption, natural organic matter, contaminant gradients, desorption behavior, and alternative exposure pathways. MPs are therefore better conceptualized as dynamic sorbing interfaces than as uniformly effective contaminant carriers.

### 3.5 Climate-sensitive hydrology generates episodic exposure that steady-state sampling can miss

Hydrological extremes demonstrate that environmental MP exposure can vary by orders of magnitude over very short periods. During a storm in the Cooks River estuary, Australia, Hitchcock (2020) measured an increase in MP abundance from approximately 400 particles m^−^^3^ before the storm to 17,383 particles m^−^^3^ following heavy rainfall—an increase of more than 40-fold. MP abundance was positively associated with antecedent rainfall, indicating mobilization of particles accumulated within the urban catchment. Such short-lived exposure pulses may be poorly represented by monitoring programs based on low-frequency sampling. The same phenomenon has been observed at much larger spatial scales during extreme storms. In Sagami Bay, Japan, Nakajima et al. (2022) measured 101,762 ± 13,213 MP pieces km^−^^2^ three days before Typhoon Faxai and 23,032,769 ± 8,372,347 pieces km^−^^2^ one day after passage of the typhoon, corresponding to a 226-fold increase. The combined mass of micro- and mesoplastics increased approximately 1,309-fold, from 0.070 ± 0.035 to 91.6 ± 30.9 kg km^−2^. By three days after the typhoon, particle abundance and mass had returned close to pre-event values. The rapid rise and subsequent decline indicate that extreme events can simultaneously mobilize terrestrial particles, create acute exposure pulses, and export material from coastal systems. These event studies require careful interpretation in the context of climate change. They establish that MP transport is highly sensitive to rainfall, flooding, runoff, and storm-driven circulation; they do not, by themselves, demonstrate that anthropogenic climate change has already produced a specific increase in environmental MP concentrations. The stronger inference is mechanistic: changes in the frequency and intensity of extreme hydrological events can modify processes already known to mobilize MPs. Maintaining this distinction between process sensitivity and climate attribution avoids converting plausible future interactions into unsupported causal claims. Collectively, these studies demonstrate that MP exposure can change substantially across source, transport, aging, chemical conditioning, and hydrological stages, with the direction and magnitude of change depending on particle properties and environmental context. Table 1 represents the evidence linking these processes to changes in MP mobility, transformation, contaminant interactions, and environmental exposure.

**Table 1:** Processes controlling environmental transformation and exposure of microplastics.

Process/driver	Representative evidence	Mechanistic interpretation	Implication for exposure and risk	Limitation	References
Tire-road-wear generation	Estimated tire-wear emissions ranged from 0.23 to 4.7 kg capita^−1^ year^−1^, with a global average of ~0.81 kg capita^−1^ year^−1^	Use-phase emissions can constitute substantial inputs of compositionally complex particles containing elastomers, fillers, metals, and additives	Source identity influences subsequent chemistry, mobility, and toxicity; tire particles should not be treated as equivalent to pristine polymer beads	National estimates depend on vehicle use, tire properties, road conditions, and emission factors	Kole et al., 2017
Textile-fiber release	A single garment released >1,900 fibers per washing cycle under tested conditions	Domestic laundering directly generates high-aspect-ratio particles with transport properties different from spheres	Source-related morphology can strongly affect wastewater entry, suspension, and atmospheric or aquatic transport	Release varies with fabric construction, garment age, washing conditions, and analytical method	Browne et al., 2011
Atmospheric shape-dependent transport	Settling velocities of MP fibers were up to 76% lower than those of volume-equivalent spheres	High-aspect-ratio fibers remain airborne longer and can travel farther than spherical particles of equivalent volume	Models assuming spherical particles may underestimate atmospheric residence and long-range transport	Transport remains dependent on fiber dimensions and meteorology	Tatsii et al., 2024
Atmospheric deposition	Fiber deposition ranged from 2 to 355 particles m^−2^ day^−1^; mean values were 110 ± 96 and 53 ± 38 particles m^−2^ day^−1^ at urban and less-urbanized sites, respectively	Atmospheric fallout can redistribute fibrous particles between urban surfaces, soils, and waters	Deposition represents an important cross-compartment exposure pathway	Only ~29% of characterized fibers were entirely or partly synthetic, showing that morphology alone cannot establish plastic identity	Dris et al., 2016
Biofouling-mediated vertical transport	Under one modeled 8-h-light/16-h-dark scenario, sinking of a 1-mm buoyant PE particle began only after >116 days, whereas greater light availability accelerated biofilm-driven sinking	Biofilm growth changes effective density and can produce sinking, resurfacing, or vertical oscillation	Biofouling redistributes exposure vertically rather than necessarily creating permanent sequestration	Process-based model; timescales are not universal field values	Kooi et al., 2017
Soil bioturbation	Earthworms redistributed PE particles from the soil surface to approximately 10 cm depth within 21 days; particle size and earthworm presence significantly affected distribution (*P* < 0.001)	Biological activity can transport MPs through soils independently of passive pore-water movement	Bioturbation can expose deeper soil horizons, roots, and microbial communities to MPs	Controlled soil experiment using defined PE particle sizes	Rillig et al., 2017
Accelerated photo-weathering	Pollutant uptake increased by >two-fold after 10 days of accelerated weathering	Oxidation and surface roughening can increase dispersibility and interfacial reactivity before polymer removal occurs	Aging may increase contaminant interactions even when polymer mass remains present	Accelerated laboratory aging cannot be converted directly into environmental years	Al Harraq et al., 2022
Fragmentation to smaller particles	For PS, ~92,465 particles mL^−1^ were detected in the 2–60 μm fraction after 112 days vs. 2,147 mL^−1^ in controls; ~6.4 × 10^8^ particles mL^−1^ occurred in the 30–2,000 nm fraction after 84 days	Polymer deterioration can greatly increase particle number while transferring mass toward smaller size fractions	Apparent “degradation” may increase exposure to micro- and nanoscale particles rather than eliminate plastic	Laboratory generation rates should not be extrapolated directly to environmental production rates	Lambert and Wagner, 2016
Aging-enhanced metal adsorption	After 30 days aging, Cd(II) adsorption increased by 40.61% for PE and 25.49% for PLA; Cr(VI) adsorption increased by 37.50% and 69.29%, respectively	Oxidation and formation of new surface functional groups alter contaminant affinity	Weathered MPs can behave chemically differently from pristine particles	Magnitude depends on polymer, contaminant, solution chemistry, and aging protocol	Zhang et al., 2024
Field accumulation of POPs	PAHs and PCBs accumulated on five polymer types during deployment for up to 12 months in San Diego Bay	Environmental plastics can acquire persistent organic contaminants over realistic residence times	Confirms environmental contaminant loading of plastic surfaces	Sorption alone does not establish that MPs dominate organism exposure	Rochman et al., 2013a,b
Relative importance of MP-bound hydrophobic contaminants	Model estimate: ~98.3% of HOCs resided in water, whereas only ~0.0002% was associated with plastic in the modeled ocean scenario	Strong sorption capacity does not necessarily translate into a dominant contaminant reservoir	MP-mediated chemical exposure should be evaluated relative to diet, sediment, dissolved phases, and other natural sorbents	Multimedia modeling depends on environmental assumptions and equilibrium/kinetic parameterization	Koelmans et al., 2016
Antibiotic sorption	For PA, freshwater distribution coefficients across tested antibiotics ranged from approximately 7.36–756 L kg^−1^	Antibiotic association is strongly dependent on polymer functionality and compound chemistry	“MPs as antibiotic vectors” cannot be generalized across all polymers or water matrices	Sorption was weaker in seawater and varied strongly among antibiotics	Li et al., 2018
Natural organic matter effect on antibiotic sorption	0.1 M NaNO_3_ reduced ciprofloxacin adsorption to PE by ~17%, while humic acid reduced adsorption by approximately 89%	Environmental organic matter and ionic conditions can strongly suppress apparent sorption capacity	Simplified laboratory water may overestimate contaminant carriage by environmental MPs	Result is condition-specific and depends on pH, DOM composition, and solution chemistry	Atugoda et al., 2020
Storm-driven mobilization	MP abundance increased from approximately 400 to 17,383 particles m^−3^ after heavy rainfall, i.e., >40-fold	Storm runoff can mobilize particles stored within urban catchments and create acute exposure pulses	Low-frequency monitoring may miss short-duration peaks in MP exposure	Single-event magnitude cannot be generalized across catchments	Hitchcock, 2020
Typhoon-driven redistribution	MP abundance increased 226-fold 1 day after a typhoon; combined micro- and mesoplastic mass increased approximately 1,309-fold, followed by rapid decline	Extreme events can rapidly mobilize, redistribute, and export accumulated plastics	Exposure can be highly episodic rather than steady state	Site- and event-specific response; does not by itself demonstrate climate-change attribution	Nakajima et al., 2022

Cd(II), cadmium(II); Cr(VI), chromium(VI); DOM, dissolved organic matter; HOCs, hydrophobic organic contaminants; MP(s), microplastic(s); NaNO3, sodium nitrate; PA, polyamide; PAHs, polycyclic aromatic hydrocarbons; PCBs, polychlorinated biphenyls; PE, polyethylene; PLA, polylactic acid; POPs, persistent organic pollutants; PS, polystyrene.

## 4 Plastisphere ecology and microbially mediated transformation

Environmental conditioning described above creates surfaces on which microbial attachment can proceed, converting an aged particle–chemical interface into a biologically active system. The term plastisphere denotes the microbial assemblages associated with plastic surfaces, but its ecological significance extends beyond the presence of attached cells. Zettler et al. (2013) demonstrated that marine plastic debris supported structured microbial communities distinct from surrounding seawater, including bacteria, phototrophs, grazers, and other eukaryotes. Subsequent studies have shown that these communities can modify particle surface properties, contaminant partitioning, nutrient transformations, antimicrobial-resistance dynamics, and potentially polymer transformation. The critical question is therefore not simply whether MPs are colonized—colonization is widespread—but whether the resulting biofilm produces functions that measurably alter environmental fate or risk. The mechanistic progression from surface conditioning and community assembly to functional transformation is summarized in Figure 2.

**Figure 2:**
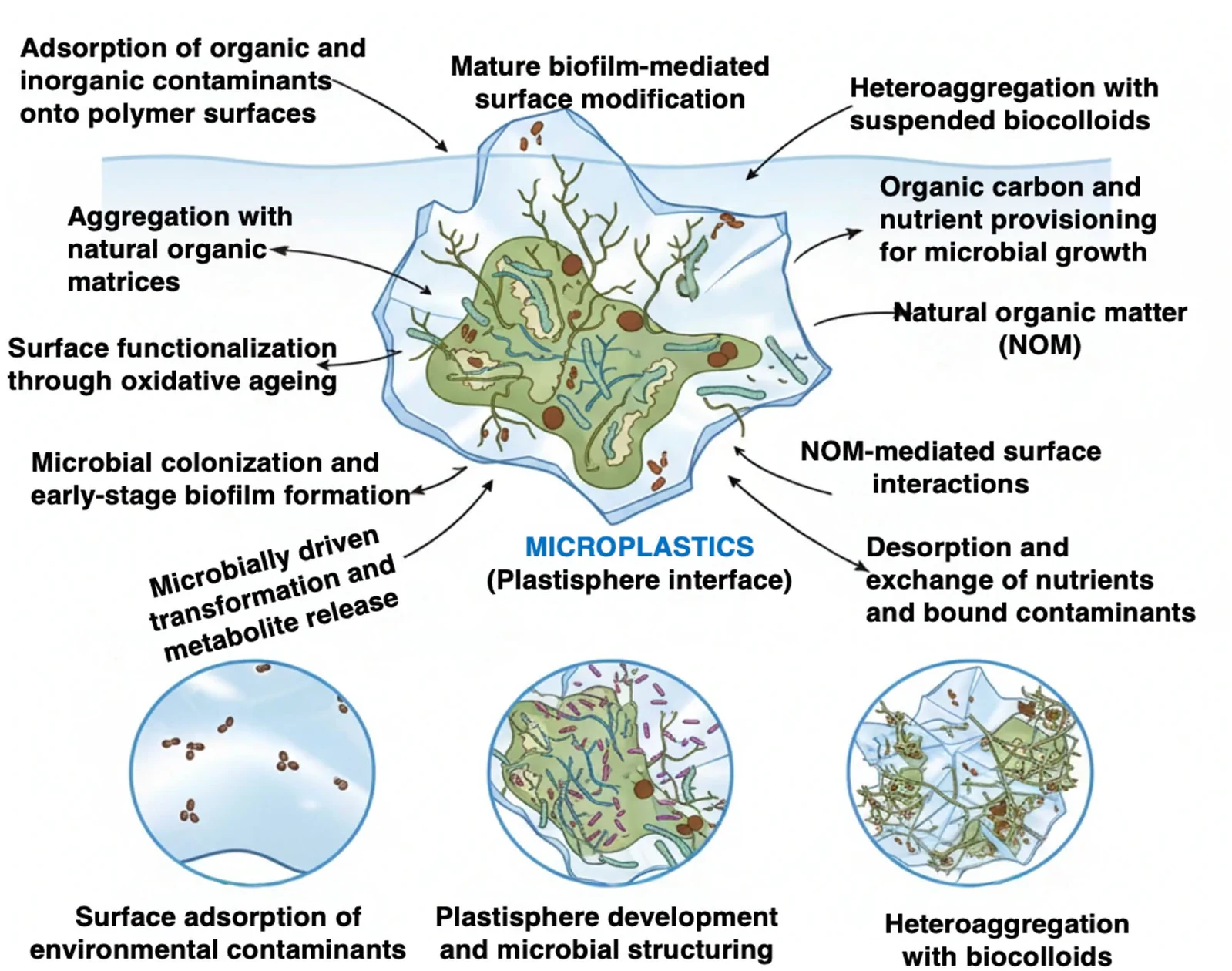
Formation and functional consequences of the plastisphere interface on environmentally conditioned microplastics. Environmental aging, adsorption of organic and inorganic contaminants, interaction with natural organic matter (NOM), and early microbial colonization progressively transform microplastic surfaces into biologically active plastisphere interfaces.

### 4.1 Plastisphere formation and microbial community assembly

Initial colonization is governed by the interaction between the conditioned particle surface and the microorganisms available in the surrounding environment. Adsorbed organic molecules provide a conditioning layer within which pioneer microorganisms attach, proliferate, and produce extracellular polymeric substances (EPS). EPS subsequently increases surface heterogeneity and generates microgradients in oxygen, nutrients, pH, and redox conditions, allowing a progressively more complex biofilm to develop. The resulting assemblage is therefore not a simple taxonomic coating on plastic but a spatially structured microbial habitat. Absolute-abundance measurements illustrate how strongly MPs can concentrate microorganisms relative to their surroundings. In municipal sewer systems, Ye et al. (2024) reported that microbial colonizer loads associated with MPs were 8.7–56.0 times higher at field scale and 1.26–5.62 times higher in laboratory experiments than corresponding reference communities. Interestingly, this increase in microbial abundance did not imply greater diversity: plastisphere communities were generally less diverse than surrounding sediment communities, indicating that plastic surfaces concentrated a subset of environmentally available organisms rather than passively accumulating all taxa. Under aerobic conditions, colonizer loads were additionally 1.71 times higher than under anaerobic conditions, demonstrating that redox state can materially influence biofilm development.

Surface chemistry also modifies early colonization, although polymer identity is not invariably the dominant ecological filter. During a 6-week marine incubation, Dussud et al. (2018) observed successive phases of initial attachment, biofilm growth, and maturation on both conventional and nominally biodegradable polymers. Biodegradable poly(3-hydroxybutyrate-co-3-hydroxyvalerate) and pro-oxidant-containing materials supported greater abundances of metabolically active and polymer-specific bacterial groups than conventional polyethylene under the tested conditions. However, hydrocarbonoclastic bacteria occurred across several polymer types, suggesting that enrichment of putative hydrocarbon-utilizing taxa alone cannot establish polymer degradation. Environmental context can override polymer effects. Oberbeckmann et al. (2018) exposed polyethylene, polystyrene, and wooden particles for 2 weeks along a gradient from coastal Baltic seawater to wastewater-affected freshwater conditions. Plastic-specific assemblages developed predominantly under the lower-nutrient, higher-salinity conditions, whereas the surrounding environment explained much of the overall community differentiation. The study found no general enrichment of potential pathogens on polyethylene or polystyrene, although bacteria commonly associated with antimicrobial resistance were abundant on MPs within the wastewater-treatment environment. These results are important because they challenge the concept of a universal “plastic microbiome”: plastisphere composition emerges from interactions among polymer characteristics, aging state, nutrient conditions, salinity, inoculum composition, redox state, and colonization time rather than from polymer identity alone.

### 4.2 Biofilm development changes particle properties and contaminant interactions

Accumulation of cells and EPS increases effective surface area, introduces charged and chemically reactive functional groups, traps dissolved and particulate organic matter, and creates new binding domains for metals and organic contaminants. The resulting sorption behavior may therefore reflect the biofilm as much as the underlying polymer. Bhagwat et al. (2021) directly quantified this effect using naturally aged, biofilm-coated polyethylene, polypropylene, nylon, and polyester microfibers. Relative to corresponding virgin fibers, biofilm-coated materials adsorbed 4%−25% more lead and 20%−85% more perfluorooctane sulfonate (PFOS), with the magnitude varying among polymers. Surface-area measurements showed the sequence polypropylene, polyethylene, nylon, then polyester after biomass accumulation. These results demonstrate that environmental aging and microbial growth can substantially change contaminant partitioning, but they also show why a single “biofilm enhancement factor” is inappropriate: the response depended on both contaminant properties and polymer–biofilm characteristics.

Microbial colonization can also alter contaminants after they reach the interface. This possibility should not, however, be equated with automatic detoxification. Wang et al. (2022) compared tetracycline transformation in systems containing polylactic acid, polyvinyl chloride, or quartzite biofilms. After 28 days, approximately 24% of tetracycline was biodegraded in the quartzite system, whereas only 17.3 and 16.7% were degraded in the polylactic acid- and polyvinyl chloride-containing systems, respectively. Metagenomic analysis indicated greater abundance of potential tetracycline-degrading microorganisms and associated metabolic genes on quartzite than within the plastisphere. Thus, despite the ability of MPs to concentrate antibiotics, their biofilms were less effective at degrading tetracycline than a natural mineral-associated biofilm under these conditions. This counterexample is important for interpreting contaminant–plastisphere interactions. Greater contaminant accumulation on a biofilm-coated MP does not necessarily produce greater microbial transformation, and microbial transformation itself does not necessarily produce lower toxicity. Substrate availability, diffusion through EPS, community composition, redox microzones, contaminant concentration, and availability of alternative carbon sources can determine whether sorbed compounds are metabolized, retained, released, or transformed into intermediate products. Consequently, the plastisphere should be considered a microreactor with context-dependent function, not uniformly as either a contaminant sink or a detoxification surface.

The relationship is also bidirectional: associated chemicals select the microbial community. Wang et al. (2022) found greater antibiotic resistance gene (ARG) carriage in plastisphere communities than in quartzite-associated biofilms, while biodegradable polylactic acid supported more biomass and ARGs than non-biodegradable polyvinyl chloride. The authors proposed that stronger contaminant association and availability of polymer- or biofilm-derived carbon altered selective pressure and metabolic strategy. Chemical conditioning therefore changes microbial ecology at the same time that microbial activity changes contaminant fate.

### 4.3 Plastisphere biofilms as hotspots for antimicrobial-resistance exchange

One of the most microbiologically important consequences of surface-associated growth is the creation of high-cell-density microenvironments in which bacteria are physically retained in close proximity. Such conditions can facilitate cell–cell contact, extracellular DNA retention, plasmid exchange, and other processes relevant to horizontal gene transfer. MPs can additionally co-concentrate antibiotics, metals, disinfectant residues, and other selective agents, creating conditions in which resistance determinants may have an ecological advantage. Direct experimental evidence demonstrates that the biofilm state itself can increase conjugative gene transfer. Zhou et al. (2024) compared ARG transfer between suspended bacteria and biofilms formed on different MPs in an estuarine system. Conjugative-transfer frequencies were 7.2–19.6 times higher in MP biofilms than among suspended bacteria, with PE-associated biofilms producing the strongest enhancement. Mechanistic analyses linked the effect to increased bacterial density together with elevated expression of outer-membrane proteins, conjugative-pilus-associated functions, and DNA replication and transfer systems. This moves the argument beyond simple ARG detection: under the experimental conditions, the plastisphere actively altered the probability of horizontal transfer.

The environmental significance nevertheless depends strongly on the surrounding microbial and chemical setting. Oberbeckmann et al. (2018) did not observe general enrichment of potential pathogens on polyethylene or polystyrene across their Baltic-to-freshwater gradient, despite enrichment of bacterial groups associated with resistance in the wastewater-treatment setting. Thus, plastic surfaces should not be described universally as pathogen-enrichment substrates. Wastewater, aquaculture, hospital effluent, urban rivers, and other environments with high bacterial density and antimicrobial selection pressure are more plausible locations for resistance-related plastisphere effects than oligotrophic waters with low pathogen loading.

Full-scale treatment data support this conditional interpretation. Bydalek et al. (2023) followed MP-associated biofilms through a conventional wastewater-treatment system coupled to a constructed wetland. The combined treatment reduced pathogen and AMR loads associated with MPs by up to two orders of magnitude (>99%), demonstrating that plastisphere-associated risk can be substantially attenuated. Nevertheless, *Aeromonas, Klebsiella*, and *Streptococcus* remained among pathogenic genera associated with AMR, and the class 1 integron marker intI1 persisted as an indicator of residual resistance potential. When sewage-conditioned MPs entered the wetland directly rather than after conventional treatment, attenuation was less effective. The persistence of attached organisms through treatment is also evident from Kruglova et al. (2022), who found that core bacterial groups remained strongly associated with MPs throughout municipal wastewater treatment and were subsequently detectable on particles leaving with treated effluent. Potentially pathogenic taxa occurred at multiple treatment stages, with many retained on effluent MPs. The implication is not that every environmental MP carries viable pathogens, but that particle-associated transport can protect and export microbial assemblages that might otherwise have different settling, predation, or treatment-removal trajectories.

Together, these studies distinguish three separate propositions that should not be conflated: MPs can carry ARGs, plastisphere conditions can select for resistant microorganisms, and biofilm growth can increase horizontal transfer rates. Evidence for all three exists, but their magnitude depends strongly on environmental context. This distinction provides a more defensible microbial-risk framework than treating ARG detection alone as proof that MPs universally amplify antimicrobial resistance.

### 4.4 Microplastics alter microbial nitrogen, phosphorus, and carbon cycling

The ecological importance of the plastisphere extends beyond contaminant and resistance dynamics because attached microorganisms participate in biogeochemical transformations. Plastic surfaces introduce both a physical habitat and, for some materials, a potential carbon source or additive source. They can also modify oxygen penetration, organic-matter retention, and substrate gradients. These changes may restructure microbial functional networks even when the polymer backbone itself is not substantially biodegraded. Seeley et al. (2020) demonstrated strong polymer-specific effects in a 16-day salt-marsh sediment experiment containing polyethylene, polyvinyl chloride, polyurethane foam, or polylactic acid. Polyurethane foam - and polylactic acid -amended sediments showed increased nitrate and nitrite, reduced ammonium, and elevated ammonia monooxygenase subunit A gene *(amoA)* abundance consistent with enhanced nitrification, whereas polyvinyl chloride contained almost no detectable nitrate or nitrite and exhibited the lowest amoA abundance. Potential denitrification was significantly greater in polylactic acid and polyurethane foam treatments than in the control (*P* < 0.05), while polyvinyl chloride produced the lowest rate among plastic treatments. The same nominal category—“MP exposure”—therefore produced opposite effects on nitrogen transformation depending on polymer chemistry. The Seeley et al. (2020) experiment also illustrates why microbial functional markers require careful interpretation. Relative abundances of cytochrome cd1 nitrite reductase gene *(nirS)* and copper-containing nitrite reductase gene *(nirK)* did not map perfectly onto measured denitrification rates; the control contained comparatively high denitrification-gene abundances but substantially lower activity than polylactic acid and Polyurethane foam treatments. Substrate availability, particularly nitrate/nitrite and organic carbon, therefore constrained function independently of gene abundance. This discrepancy is highly relevant to plastisphere studies relying solely on metagenomic prediction: functional genes indicate metabolic potential, whereas measured fluxes establish ecosystem process rates.

Freshwater experiments reach a similar conclusion. Chen et al. (2020) first colonized polypropylene surfaces in a pond for 30 days and then introduced the mature biofilms into environmentally relevant microcosms. MP-associated biofilms accelerated ammonia oxidation, nitrite oxidation, and denitrification and temporarily accumulated phosphorus while increasing alkaline-phosphatase activity. As mature biofilms subsequently disintegrated, nitrogen and phosphorus were released back to the water, demonstrating that plastisphere effects can change sign during biofilm succession. Nutrient sequestration by a growing biofilm may therefore become nutrient release during senescence rather than representing permanent removal.

Metabolic-network analyses suggest that these effects extend beyond nitrogen alone. In municipal sewer systems, Ye et al. (2024) identified sediment-associated plastisphere networks oriented toward hydrolysis/fermentation, hydrogen-producing acetogenesis, methanogenesis, and denitrification, whereas wastewater-associated MPs exhibited stronger niche partitioning and competitive interactions. These functional predictions are consistent with redox-stratified biofilms but should be interpreted as potential metabolic organization unless accompanied by direct process-rate measurements. Collectively, these findings demonstrate that MPs can modify biogeochemical cycling by altering microbial habitat structure, substrate availability, and community organization. The direction of response is nevertheless neither uniform nor inherently adverse. Enhancement of denitrification may reduce reactive nitrogen under one set of conditions, while shifts toward incomplete denitrification or altered carbon metabolism could generate different ecosystem consequences under another. Determining ecological significance therefore requires rate measurements and mass balances, rather than taxonomic shifts alone.

### 4.5 Microbial colonization, biodeterioration, and true polymer biodegradation must be distinguished

Perhaps the most important conceptual distinction in interpreting plastisphere studies is that biofilm formation is not evidence of polymer biodegradation. Surface attachment, EPS production, changes in wettability, localized pitting, and enrichment of taxa with putative hydrocarbon-degrading capabilities demonstrate biological interaction with a plastic surface, but they do not establish cleavage of the polymer backbone or utilization of polymer-derived carbon. The evidence connecting plastisphere formation to environmental transformation, contaminant interactions, antimicrobial resistance, biogeochemical cycling, and polymer degradation is summarized in Table 2. Nauendorf et al. (2016) provide a particularly clear experimental demonstration. PE and commercially biodegradable plastic were incubated in oxic and anoxic marine sediments for 98 days. The nominally biodegradable material supported approximately five-fold greater aerobic colonization and eight-fold greater anaerobic colonization than polyethylene. Despite this substantial difference in microbial abundance, neither material showed evidence of biodegradation based on mass, surface, and chemical analyses. High colonization intensity therefore predicted neither polymer breakdown nor environmental disappearance. This finding helps reinterpret observations of plastisphere taxa associated with hydrocarbon metabolism. Dussud et al. (2018) detected hydrocarbonoclastic bacteria during colonization of multiple plastic types, but the occurrence of these organisms may reflect utilization of adsorbed dissolved organic matter, additives, oligomers, EPS-derived carbon, or other compounds at the interface rather than direct metabolism of the high-molecular-weight polymer backbone. Functional inference from taxonomy alone therefore risks assigning “plastic-degrading” activity without demonstrating its carbon source. Increased microbial activity can arise because polymer-associated components or hydrolysis products modify carbon availability without establishing substantial polymer mineralization. Conversely, a surface may undergo measurable chain scission without significant microbial assimilation. For this reason, evidence of microbially mediated polymer transformation should be interpreted as a sequence of increasingly stringent endpoints: (i) colonization, (ii) biodeterioration/surface modification, (iii) polymer-chain cleavage, (iv) oligomer/monomer release, (v) assimilation of polymer-derived carbon, and (vi) mineralization or verified conversion into cellular/products carbon. Only the later steps demonstrate true degradation of polymer carbon. This hierarchy is also essential for the valorization discussion later in the manuscript, where microbial growth on plastic cannot be treated as evidence that environmental MPs can be efficiently converted into useful products.

**Table 2:** Plastisphere-mediated environmental transformation and microbial risk.

Microbial process	Experimental evidence	Main result	Mechanistic interpretation	Critical limitation	References
Microbial colonization	Field and laboratory sewer plastisphere	MP-associated microbial loads increased 8.7–56.0 × in field samples and 1.26–5.62 × in laboratory tests; aerobic colonizer load was 1.71 × anaerobic load	MPs can strongly concentrate subsets of environmental microorganisms and create distinct interaction networks	Engineered sewer environment; magnitude cannot be generalized to natural waters	Ye et al., 2024
Biofilm-mediated contaminant sorption	Naturally aged PE, PP, nylon and polyester microfibers	Biofilm-coated fibers adsorbed 4%−25% more Pb and 20%−85% more PFOS than virgin fibers	EPS and accumulated biomass create additional reactive sorption domains	Enhancement varied strongly with polymer and contaminant	Bhagwat et al., 2021
Antibiotic transformation	PLA-, PVC- and quartzite-associated biofilms	Tetracycline degradation after 28 day: 24% quartzite, 17.3% PLA, 16.7% PVC	Plastisphere biofilms are not inherently superior contaminant-degrading communities	Single antibiotic and urban aquatic system	Wang et al., 2022
Horizontal ARG transfer	Estuarine MP biofilms vs. suspended bacteria	Conjugative ARG transfer increased 7.2–19.6 ×, with PE biofilm producing the strongest enhancement	High cell density and transfer-system expression can make mature biofilms hotspots for conjugation	Controlled donor/recipient system; environmental transfer rates may differ	Zhou et al., 2024
Pathogen/AMR attenuation during treatment	Full-scale conventional treatment plus constructed wetland	MP-associated pathogen and AMR loads decreased by up to two orders of magnitude (>99%)	Plastisphere-associated microbial risk can be attenuated substantially during treatment, but not necessarily eliminated	Treatment-specific result; intI1 and pathogenic genera remained detectable	Bydalek et al., 2023
Nitrogen-cycle alteration	PE, PVC, PUF and PLA in salt-marsh sediments for 16 days	PUF and PLA enhanced nitrification and produced denitrification rates significantly above control (*P* < 0.05); PVC inhibited both pathways	Polymer chemistry can alter microbial process rates in opposite directions	Short microcosm study and relatively high experimental amendment	Seeley et al., 2020
Freshwater nutrient cycling	Pond-colonized PP biofilms, 30-day precolonization	Biofilms accelerated ammonia oxidation, nitrite oxidation and denitrification; P was initially retained and later released during biofilm disintegration	Plastisphere effects can change during biofilm maturation and senescence	Process magnitude requires broader field validation	Chen et al., 2020

AMR, antimicrobial resistance; ARG, antibiotic resistance gene; EPS, extracellular polymeric substances; *intI1*, class 1 integron integrase gene; MP(s), microplastic(s); P, phosphorus; Pb, lead; PE, polyethylene; PFOS, perfluorooctane sulfonate; PLA, polylactic acid; PP, polypropylene; PUF, polyurethane foam; PVC, polyvinyl chloride.

## 5 Ecotoxicological consequences across biological scales

The ecotoxicity of MPs emerges from interactions among particle dimensions and morphology, environmental aging, associated chemicals, microbial conditioning, exposure duration, and organism physiology. Consequently, biological responses cannot be assigned to the polymer alone. The same particle may act initially as a physical stressor, subsequently induce oxidative or inflammatory responses after uptake, and simultaneously alter exposure to associated chemicals or microorganisms. These mechanisms operate across hierarchical levels extending from cellular perturbation to tissue injury, altered growth and reproduction, food-web transfer, and potentially ecosystem-level change. Importantly, responses at one level do not automatically predict effects at another: an oxidative-stress biomarker does not by itself demonstrate impaired fitness, and trophic transfer does not establish biomagnification. The evidence considered below is therefore organized according to increasing biological scale and strength of ecological inference, as summarized conceptually in Figure 3.

**Figure 3:**
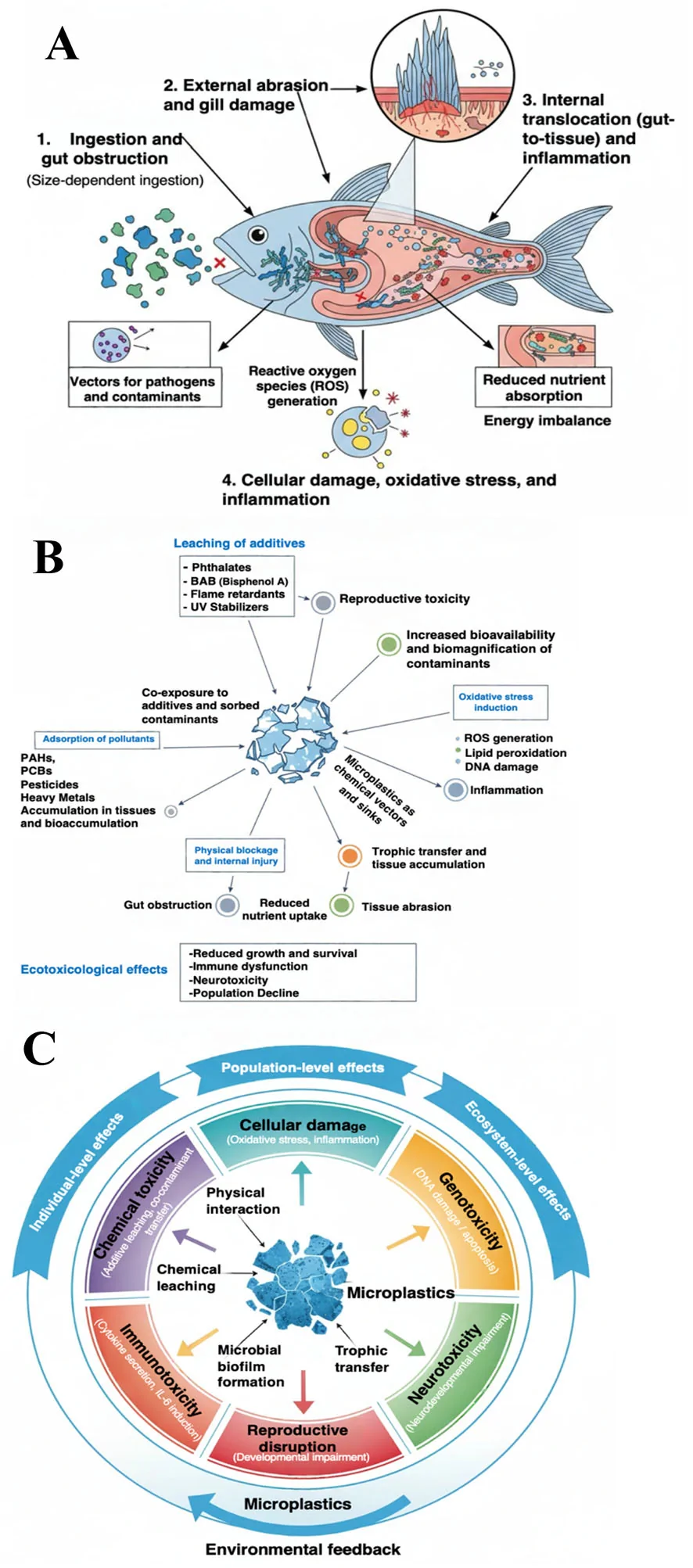
Ecotoxicological consequences of microplastics across biological scales. **(A)** Representative organism-level pathways of microplastic injury, including ingestion, gut obstruction, external abrasion, gill damage, internal translocation, oxidative stress, inflammation, reduced nutrient absorption, and energy imbalance. **(B)** Conceptual pathways through which microplastics and associated additives or sorbed contaminants contribute to ecotoxicological effects. **(C)** Hierarchical organization of microplastic effects across biological scales, linking physical interaction, chemical leaching, microbial biofilm formation, and trophic transfer to cellular responses, individual-level effects, and broader population- and ecosystem-level consequences.

### 5.1 Particle uptake, physical interference, and size-dependent tissue distribution

Ingestion represents the first biological interaction for many aquatic and terrestrial organisms, but retention and translocation depend strongly on particle dimensions. Browne et al. (2008) exposed *Mytilus edulis* to 3.0- and 9.6-μm PS microspheres and demonstrated transfer from the digestive tract into the circulatory system within approximately 3 days. Particles remained detectable in haemolymph for more than 48 days, and smaller 3-μm particles entered the circulatory compartment more efficiently than the larger particles. Notably, persistent particle presence did not produce clear adverse effects in the immune and oxidative endpoints measured in that experiment. This distinction is important: tissue translocation establishes internal exposure, but it does not by itself establish toxicity.

Size dependence becomes clearer in vertebrates. Lu et al. (2016) exposed zebrafish to polystyrene particles and found that 5-μm particles accumulated in gills, gut, and liver after 7 days, whereas 20-μm particles were detected in gills and gut but not liver. Exposure to 5-μm MPs and 70-nm PS particles was additionally associated with hepatic inflammation, lipid accumulation, increased superoxide dismutase and catalase activity, and disruption of lipid and energy metabolism. The divergence between 5- and 20-μm particles demonstrates that nominal polymer identity is less informative than the combination of polymer and size when predicting internal exposure. Physical impacts are not necessarily monotonic with concentration or transferable among polymer types. In a recent 28-day soil experiment, Liu C. et al. (2026) and Liu X. et al. (2026) exposed *Eisenia fetida* to polyethylene, polypropylene, and biodegradable poly(butylene adipate-co-terephthalate) (PBAT) MPs at 0.05%, 0.5%, and 2% w/w. Exposure to 2% PP produced 16.7% mortality, whereas 0.05% PP increased growth by 42.1% relative to controls. Despite this apparent low-dose stimulation, reproductive efficiency was disrupted across treatments, and histological injury and oxidative stress emerged at higher concentrations. PBAT generated particularly strong cellular responses, including elevated 8-hydroxy-2′-deoxyguanosine. These results caution against two common generalizations: concentration–response relationships for particulate stress are not necessarily linear, and classification as “biodegradable” does not establish biological safety. Particle uptake therefore represents an exposure gateway rather than a toxic endpoint. Whether ingestion progresses to injury depends on retention time, ability to cross epithelial barriers, particle shape, biological clearance, nutritional status, and tissue susceptibility. Studies based on smooth spherical particles may consequently underestimate abrasion by irregular environmental fragments while simultaneously overestimating long-term retention if experimental concentrations overwhelm normal clearance mechanisms.

### 5.2 Cellular and molecular responses

Oxidative stress is among the most frequently reported molecular responses to MP exposure, but its interpretation requires caution. Increased reactive oxygen species or activation of antioxidant enzymes indicates disturbance of cellular redox regulation; it does not necessarily indicate irreversible injury. In the zebrafish experiment of Lu et al. (2016), increased superoxide dismutase and catalase activities occurred together with hepatic inflammation, lipid accumulation, and metabolomic disruption, providing stronger evidence of an adverse response than antioxidant-enzyme induction alone. Such multilevel concordance is important because individual biomarkers can also represent adaptive compensation.

Genotoxicity provides a second mechanistic link between particle exposure and longer-term biological effects. Avio et al. (2015) exposed *Mytilus galloprovincialis* to PE and PS MPs with and without adsorbed pyrene and demonstrated particle localization in haemolymph, gills, and digestive tissues together with altered lysosomal stability, antioxidant responses, neurotoxicity-related biomarkers, gene-expression profiles, and genotoxic endpoints. Pyrene-loaded MPs increased chemical bioavailability to mussels, showing that the biological response reflected a combined particle–chemical exposure rather than a polymer effect in isolation. Such experiments provide mechanistic evidence for mixture toxicity but should not be interpreted as demonstrating that MP-associated chemicals dominate contaminant exposure under all environmental conditions.

Importantly, biological responses to mixtures are not consistently additive or synergistic. Mijošek Pavin et al. (2026) exposed *M. galloprovincialis* to 1 mg L^−1^ polypropylene (~40 μm), 50 μg L^−1^ pyrene, or both for 7 and 14 days. DNA damage increased significantly following polypropylene or pyrene exposure individually, yet remained near control levels under combined exposure, while other physiological and antioxidant endpoints showed evidence of stress. Thus, co-exposure attenuated one genotoxic endpoint while altering other responses. This recent result illustrates why terms such as “amplified toxicity” should not be applied automatically to MP–pollutant mixtures: antagonistic, additive, and synergistic interactions can occur simultaneously across different tissues and biomarkers. The same interpretive caution applies to transcriptomic and metabolomic responses. Changes in genes involved in oxidative defense, apoptosis, inflammation, DNA repair, energy metabolism, or immune pathways provide valuable mechanistic information, but statistical pathway enrichment does not necessarily translate into impaired growth, survival, or reproduction. Stronger adverse-outcome inference arises when molecular changes co-occur with tissue pathology and organism-level effects and when a biologically plausible sequence connects these levels.

### 5.3 Chemical co-exposure can increase, decrease, or redistribute toxicity

MP-associated contaminants can modify biological responses, but the magnitude of this vector effect is strongly exposure dependent. Lu et al. (2018) exposed zebrafish to cadmium together with MPs for 3 weeks and found that 20 and 200 μg L^−1^ MP treatments increased cadmium accumulation in the liver by approximately 46 and 184%, respectively. Corresponding increases were approximately 10 and 25% in the gut and 9 and 46% in the gills. Co-exposure additionally increased oxidative damage and inflammation. These tissue-specific differences demonstrate that an MP-mediated effect on metal toxicokinetics may be substantially greater in one organ than another and therefore cannot be inferred from whole-organism exposure alone. Earlier benthic experiments also illustrate dose-dependent reversals. Besseling et al. (2013) exposed *Arenicola marina* to polystyrene MPs equilibrated with naturally contaminated sediment. At a low polystyrene concentration of 0.074% dry weight, polychlorinated biphenyl (PCB) bioaccumulation increased by approximately 1.1–3.6-fold, depending on congener, whereas higher MP concentrations did not produce proportionally larger PCB burdens and in some cases reduced accumulation relative to the low-dose treatment. Feeding activity was reduced at 7.4% polystyrene dry weight. The study therefore demonstrated both particle effects on organism fitness and chemical-transfer effects, but also showed that increasing plastic concentration does not necessarily increase contaminant uptake monotonically. This complexity is reinforced by environmentally realistic exposure modeling and experimentation showing that the direction of vector effects depends on contaminant fugacity, dietary exposure, sediment or dissolved phases, ingestion and egestion, and desorption kinetics. Consequently, evidence that MPs adsorb a chemical should be separated from evidence that ingestion increases the organism's total internal dose. A further issue is whether chemicals originate from the surrounding environment or from the plastic itself. In an experimental dietary study, Rochman et al. (2013b) exposed Japanese medaka to virgin polyethylene or polyethylene that had accumulated contaminants in San Diego Bay. Fish consuming environmentally exposed polyethylene accumulated associated chemicals and developed more pronounced hepatic stress and pathology than controls; virgin polyethylene also generated biological responses, but they were less severe. The comparison indicates that environmental conditioning can modify the hazard of a nominally identical polymer and provides direct support for evaluating environmentally transformed particles rather than pristine plastic alone.

### 5.4 Chronic exposure reveals reproductive and multigenerational effects that acute tests can miss

Chronic studies are particularly important because environmentally persistent particles can generate sustained sublethal pressure without causing immediate mortality. Reproduction is one of the clearest examples. Sussarellu et al. (2016) exposed adult Pacific oysters (*Crassostrea gigas*) to 2- and 6-μm virgin polystyrene particles at 0.023 mg L^−1^ for 2 months during the reproductive cycle. Exposed oysters showed a 38% reduction in oocyte number, a 5% reduction in oocyte diameter, and a 23% reduction in sperm velocity. Effects extended to the next generation: D-larval yield decreased by 41%, while subsequent larval development declined by approximately 18%. Thus, modest changes in adult physiological state translated into measurable reproductive and offspring consequences.

Long-term fish studies similarly demonstrate that absence of mortality does not imply absence of biological cost. Zhu et al. (2020) fed Japanese medaka diets containing 500, 1,000, or 2,000 μg g^−1^ of 10-μm polystyrene for 10 weeks during maturation. No significant mortality, growth impairment, or overt developmental delay occurred, and particles were not detected in internal organs beyond the gastrointestinal tract. Nevertheless, females showed a dose-dependent reduction in egg production, and histological examination identified swollen enterocytes in exposed fish. This separation between normal survival/growth and impaired reproduction illustrates why chronic ecotoxicological assessment cannot rely on mortality as the principal endpoint.

Multigenerational *Daphnia magna* studies reveal an additional interaction between particle stress and ecological context. Schür et al. (2020) exposed daphnids to irregular secondary polystyrene particles < 63 μm over four generations under food limitation. At 10,000 particles mL^−1^, the population became extinct within one generation, whereas 2,000 particles mL^−1^ caused extinction within four generations; comparable concentrations of natural kaolin did not generate the same multigenerational toxicity. These concentrations are high relative to most current environmental measurements, so the results should not be interpreted as field-effect thresholds. Their importance lies instead in demonstrating that irregular plastic particles can produce particle-specific effects that emerge across generations and interact strongly with nutritional stress.

Environmental aging can also reduce rather than enhance chronic toxicity. In a follow-up four-generation experiment using the same general *D. magna* framework, Schür et al. (2021) compared pristine irregular polystyrene with particles pre-incubated in wastewater. Exposure to 10,000 pristine particles mL^−1^ led to extinction by the third generation, whereas animals exposed to the same concentration of wastewater-conditioned particles survived through all four generations, with survival increasing to 69 and 85% in generations F2 and F3, respectively. The authors linked the attenuation primarily to adsorption of dissolved organic matter. This result directly challenges the assumption that environmental aging inevitably increases MP toxicity and reinforces the manuscript's central argument that transformation may amplify, attenuate, or redistribute hazard. Together, chronic studies reveal endpoints that short-duration assays frequently miss: altered energy allocation, reproductive output, offspring performance, and delayed population consequences. They also demonstrate why exposure realism requires more than lowering the test concentration; food availability, particle aging, morphology, and co-stressors can fundamentally change the biological response.

### 5.5 Trophic transfer is demonstrated, but biomagnification remains unproven

Laboratory studies provide clear evidence that MPs can move from prey to predators. Farrell and Nelson (2013) exposed mussels to 0.5-μm fluorescent polystyrene particles before feeding them to the shore crab *Carcinus maenas*. Crab haemolymph contained approximately 15,033 ± 3,146 particles mL^−1^ after 24 h, declining to approximately 267 ± 120 particles mL^−1^ after 21 days. The maximum particle quantity detected in haemolymph represented only about 0.04% of the amount originally presented to the mussels, and particles declined throughout the experiment despite being detected in several tissues. Thus, trophic transfer occurred, but efficient depuration limited persistence. Transfer has also been demonstrated near the base of aquatic food webs. Setälä et al. (2014) exposed Baltic zooplankton taxa to 10-μm polystyrene microspheres and observed ingestion across mysids, copepods, cladocerans, rotifers, polychaete larvae, and ciliates. Pre-exposed zooplankton subsequently transferred microspheres to mysid shrimp within 3 h of feeding. At the same time, direct-exposure experiments showed that several zooplankton groups egested microspheres within approximately 12 h, emphasizing that food-web transfer and long-term accumulation are distinct processes. This distinction becomes critical when moving from laboratory trophic transfer to claims of biomagnification. Miller et al. (2020) synthesized 116 publications, including field data from 23,049 individual organisms representing 411 marine species. MPs were documented across multiple trophic levels, supporting widespread exposure and bioaccumulation within organisms; however, the meta-analysis found no convincing evidence for generalized biomagnification of MP particles across the marine food web. Laboratory trophic-transfer studies therefore establish pathway plausibility but do not demonstrate increasing particle burdens at successively higher trophic levels in natural ecosystems. The ecological consequence of food-web transfer may instead depend on repeated exposure, retention time, organism feeding strategy, particle size, and the transfer of associated chemicals or microorganisms. Predators that rapidly depurate particles may receive recurrent short-duration exposure without net biomagnification, whereas particles retained within prey tissues may have a higher probability of translocation during digestion. Consequently, trophic-transfer evidence should be described as food-web redistribution, unless quantitative trophic-magnification factors demonstrate increasing burdens with trophic level.

### 5.6 Human exposure is increasingly demonstrated, but causal health effects remain unresolved

Human biomonitoring has moved the discussion from theoretical exposure toward demonstrated internal occurrence, although analytical uncertainty remains substantial. Table 3 summarizes particle exposure to molecular, organismal, reproductive, trophic, and human endpoints. Jenner et al. (2022) analyzed lung tissue from 13 surgical samples using μFTIR and identified 39 MPs in 11 of 13 samples. After adjustment for procedural blanks, the mean concentration was approximately 0.69 ± 0.84 particles g^−1^ tissue. Particles were detected throughout the lung, with the highest adjusted burden in lower-lung samples. These observations support inhalation and deposition as plausible exposure pathways but do not establish that the detected particles caused pulmonary disease. Systemic exposure has also been reported. Leslie et al. (2022) used double-shot pyrolysis-GC/MS to quantify plastic polymer mass in whole blood from 22 volunteers and reported a mean summed quantifiable polymer concentration of approximately 1.6 μg mL^−1^. Polyethylene terephthalate, polyethylene, and styrene-related polymers were among the principal detected materials. Because this method reports polymer mass rather than intact particle counts and because the cohort was small, the study should be interpreted as evidence of internal plastic-polymer exposure rather than as a population-level estimate of circulating MP abundance. Placental measurements provide further evidence that particles can occur beyond primary exposure surfaces. Ragusa et al. (2021) examined six placentas by Raman microspectroscopy and identified 12 fragments, approximately 5–10 μm in size, in four placentas, including material from fetal, maternal, and chorioamniotic regions. The small sample size and analytical challenges prevent inference about prevalence or pregnancy outcomes; nevertheless, the observations demonstrate that particles within the measured size range can occur in placental tissue. More recently, Marfella et al. (2024) provided epidemiological evidence of association rather than merely detection. Among 257 patients completing follow-up after carotid endarterectomy, polyethylene was detected in plaques from 150 patients (58.4%), while polyvinyl chloride was measurable in 31 (12.1%). During a mean 33.7 ± 6.9 months of follow-up, the composite outcome of myocardial infarction, stroke, or death occurred in 20.0% of patients with detected micro-/nanoplastics compared with 7.5% of patients without detectable particles. After adjustment for cardiovascular risk factors, the reported hazard ratio was 4.53 (95% CI 2.00–10.27). This is an important observational association, but it does not demonstrate causality: residual confounding, exposure history, analytical recovery, particle source, and reverse-causation mechanisms remain unresolved.

**Table 3:** Microplastic effects across biological scales.

Biological scale/process	Model and exposure	Diagnostic result	Interpretation	Critical limitation	References
Tissue translocation	*Mytilus edulis*; 3.0- and 9.6-μm PS microspheres	Particles entered haemolymph within ~3 days and remained detectable for >48 days	Small MPs can cross digestive barriers and generate persistent internal exposure	Translocation was not accompanied by clear toxicity in the measured endpoints	Browne et al., 2008
Size-dependent organ distribution	Zebrafish (*Danio rerio*); 5- and 20-μm PS, 7 days	5 μm: gill, gut, liver; 20 μm: gill and gut only	Smaller particles have greater access to internal organs and may produce broader systemic exposure	Controlled spherical PS particles may not represent irregular environmental MPs	Lu et al., 2016
Terrestrial organism toxicity	*Eisenia fetida*; PE, PP, PBAT; 0.05%−2% w/w, 28 days	2% PP: 16.7% mortality; 0.05% PP: 42.1% increase in growth; reproductive disruption across treatments	MP responses can be polymer-specific and non-monotonic; biodegradable polymers are not necessarily biologically benign	Laboratory soil concentrations and defined particles limit direct field extrapolation	Liu C. et al., 2026; Liu X. et al., 2026
Metal co-exposure	Zebrafish; Cd + 20 or 200 μg L^−1^ MPs, 3 weeks	Cd accumulation increased by 46% and 184% in liver, 10% and 25% in gut, and 9% and 46% in gill, respectively	MPs can modify contaminant toxicokinetics in a tissue- and concentration-dependent manner	Vector importance depends on contaminant gradients, exposure medium, polymer properties, and dietary pathways	Lu et al., 2018
PCB transfer and feeding impairment	*Arenicola marina*; PS MPs in contaminated sediment	0.074% PS increased PCB accumulation by approximately 1.1–3.6 × depending on congener; feeding reduced at 7.4% PS	MP exposure can simultaneously affect organism fitness and contaminant uptake, but effects are not proportional to particle concentration	Highest PS treatments exceed concentrations in many natural sediments	Besseling et al., 2013
Particle–chemical mixture toxicity	*Mytilus galloprovincialis*; 1 mg L^−1^ PP, 50 μg L^−1^ pyrene, alone or combined	PP and pyrene individually increased DNA damage, whereas combined exposure produced DNA damage closer to control despite effects on other stress endpoints	Mixture responses may be antagonistic for one endpoint and adverse for others; synergy should not be assumed	Laboratory co-exposure with one polymer and one PAH	Mijošek Pavin et al., 2026
Reproductive toxicity and offspring effects	*Crassostrea gigas*; 2- and 6-μm PS, 0.023 mg L^−1^, 2 months	Oocyte number −38%; oocyte diameter −5%; sperm velocity −23%; D-larval yield −41%; subsequent larval development ~−18%	Chronic parental exposure can affect gametogenesis and propagate effects to offspring	Virgin spherical PS under controlled exposure	Sussarellu et al., 2016
Chronic reproductive impairment	Japanese medaka (*Oryzias latipes*); 10-μm PS in diet for 10 weeks	No significant mortality or growth impairment, but females showed dose-dependent reductions in egg production and exposed fish developed intestinal histopathology	Chronic sublethal reproductive effects can occur without overt lethality or growth suppression	Dietary concentrations and pristine PS limit direct environmental extrapolation	Zhu et al., 2020
Multigenerational toxicity	*Daphnia magna*; irregular secondary PS < 63 μm	Population extinction within 1 generation at 10,000 particles mL^−1^ and within four generations at 2,000 particles mL^−1^	Particle stress can accumulate over generations and interact strongly with food limitation	High concentrations relative to most present environmental measurements	Schür et al., 2020
Effect of environmental conditioning	*D. magna*; pristine vs. wastewater-conditioned irregular PS	Pristine 10,000 particles mL^−1^ caused population extinction, whereas animals exposed to wastewater-conditioned particles survived all four generations; survival reached 69% and 85% in F2 and F3	Environmental conditioning can attenuate rather than necessarily enhance MP toxicity	Wastewater incubation represents one specific environmental aging pathway	Schür et al., 2021
Trophic transfer	Mussel (*M. edulis*) → shore crab (*Carcinus maenas*); 0.5-μm PS	Crab haemolymph contained 15,033 ± 3,146 particles mL^−1^ at 24 h, declining to 267 ± 120 particles mL^−1^ at 21 days	Prey-mediated transfer occurs, but depuration can substantially reduce internal particle burdens	Fluorescent model PS and simplified two-level food chain	Farrell and Nelson, 2013
Planktonic food-web transfer	Zooplankton → mysid predator; 10-μm PS	MPs were transferred from pre-exposed zooplankton to predators within 3 h; several zooplankton taxa egested particles within ~12 h	Trophic transfer can occur rapidly, but ingestion does not necessarily result in long-term retention	Short-term laboratory food-web system	Setälä et al., 2014
Food-web accumulation/ biomagnification	Meta-analysis of marine organisms	116 studies, 23,049 individual organisms, 411 species	Bioaccumulation and widespread trophic exposure are supported, but generalized marine biomagnification was not demonstrated	Strong heterogeneity in sampling, particle size cut-offs, units, and detection methods	Miller et al., 2020
Human lung deposition	Human lung tissue; 13 surgical samples	39 MPs detected in 11/13 samples; blank-adjusted mean ~0.69 ± 0.84 particles g^−1^ tissue	Supports inhalation, deposition, and persistence of plastic particles in the respiratory system	Small cohort; procedural contamination and analytical size cut-offs remain important	Jenner et al., 2022
Human circulation	Whole blood from 22 donors	Mean summed quantifiable polymer concentration ~1.6 μg mL^−1^	Supports systemic occurrence of plastic-associated polymer material	Mass-based pyrolysis-GC/MS measurement does not directly provide intact particle number or morphology	Leslie et al., 2022
Human placenta	Six term placentas	12 fragments, approximately 5–10 μm, detected in four of six placentas	Supports occurrence of MPs beyond primary exposure barriers and potential maternal–fetal exposure	Very small cohort; does not establish prevalence or adverse pregnancy effects	Ragusa et al., 2021
Cardiovascular association	Carotid-plaque cohort; 257 patients completing follow-up	Clinical events occurred in 20.0% of patients with detected MNPs vs. 7.5% without; adjusted HR 4.53 (95% CI 2.00–10.27)	Provides prospective evidence of an association between vascular MNP detection and adverse cardiovascular outcomes	Observational association; residual confounding and reverse causation prevent causal inference	Marfella et al., 2024

Cd, cadmium; CI, confidence interval; DNA, deoxyribonucleic acid; F2, second filial generation; F3, third filial generation; GC/MS, gas chromatography/mass spectrometry; HR, hazard ratio; MNPs, micro- and nanoplastics; MPs, microplastics; PAH, polycyclic aromatic hydrocarbon; PBAT, poly(butylene adipate-co-terephthalate); PCB, polychlorinated biphenyl; PE, polyethylene; PP, polypropylene; PS, polystyrene.

## 6 Analytical uncertainty and cross-study comparability

The interpretation of MP occurrence, transformation, microbial interactions, and toxicity depends fundamentally on how particles are sampled, recovered, identified, and quantified. Unlike dissolved contaminants, MPs constitute a heterogeneous particulate mixture spanning several orders of magnitude in size and varying in shape, density, polymer chemistry, aging state, pigmentation, and biological or mineral coating. Consequently, an MP concentration is not an instrument-independent property of a sample: it is an operational measurement defined partly by the analytical window used to observe it. A study reporting particle ≥300 μm and another quantifying particles down to 20 μm may both be analytically valid yet describe substantially different fractions of the same environmental particle population. ISO 24187:2023 now establishes general principles for environmental MP analysis, including particle-size classification, sampling, sample preparation, and representative sample quantities, but explicitly functions as a framework of minimum requirements rather than a single universally applicable analytical protocol (ISO, 2023). Analytical uncertainty must therefore be considered part of exposure characterization itself rather than treated only as laboratory error.

### 6.1 Sampling design and size cut-off define the observable particle population

Sampling introduces the first major source of uncertainty because the amount and size distribution of particles recovered depend on sampled volume or mass, mesh or filter cut-off, sampling location, hydrodynamic conditions, and temporal variability. Large particles occur at comparatively low number concentrations and can therefore be missed when insufficient environmental material is sampled, whereas small particles may dominate particle counts but pass through coarse nets or filters. Koelmans et al. (2020) demonstrated mathematically that differences among study-specific size ranges contribute substantially to variation in reported aqueous MP concentrations; rescaling datasets to common size domains reduced this methodological non-alignment. Thus, nominal concentrations expressed in particles L^−1^ or particles m^−3^ should not be compared directly unless the underlying particle-size windows are also comparable.

The scale of this problem is evident in regulatory monitoring design. Commission Delegated Decision (EU) 2024/1441 requires MP monitoring in drinking water to analyze particles between 20 μm and 5 mm, with predefined size classes of 20–50, 50–100, 100–300, 300–1,000, and 1,000–5,000 μm. A minimum of 1,000 L of water must be sampled through a filter cascade containing 100- and 20-μm collection filters, with additional filters available for procedural blanks (European Commission, 2024). This unusually large sampling volume reflects the low particle abundance expected in drinking water rather than an analytical preference for large samples. Importantly, the 20-μm lower boundary defines the regulatory measurement window; it does not imply that particles < 20 μm are environmentally absent or toxicologically irrelevant. Sample representativeness becomes even more challenging in heterogeneous matrices. Surface waters can show short-term concentration pulses during storms, whereas sediment and soil particles are distributed unevenly through spatially heterogeneous matrices. Consequently, increasing instrumental resolution cannot compensate for a poorly representative sample. Replicate field sampling and transparent reporting of sampled volume or mass, depth, filter or net cut-off, sampling duration, and environmental conditions are therefore prerequisites for interpreting analytical precision as environmental precision.

### 6.2 Sample preparation creates polymer- and size-dependent recovery bias

Once samples are collected, density separation, digestion, filtration, transfer, and concentration procedures can selectively remove or modify particles. This is particularly important for soil, sediment, and biological matrices, where plastics constitute a small fraction of a much larger mineral or organic background. Möller et al. (2020) concluded that no single preparation strategy was optimally suited to all small MPs in soils because particle recovery changes with polymer density, matrix properties, and the separation procedure. Therefore, extraction efficiency should not be assumed from the nominal density of the separation solution alone.

Comparative experimental evidence demonstrates the magnitude of method-specific bias. Peneva et al. (2025) compared digital microscopy, fluorescence microscopy, FTIR, Raman, Py-GC/MS, and ^1^H NMR-based methods for MPs in soils. Large particles were recovered comparatively well-across approaches, whereas quantification of small biodegradable poly(butylene adipate-co-terephthalate) (PBAT)/polylactic acid particles varied substantially. Fluorescence-based analysis failed to recover 56%−93% of small PBAT/polylactic acid particles, and reliable quantification of these materials was obtained only using Soxhlet extraction followed by ^1^H NMR. Py-GC/MS showed intermediate performance and decreasing recovery with increasing soil clay content. These differences demonstrate that two analytical protocols applied to the same soil can produce different apparent polymer profiles without any true difference in environmental contamination.

Digestion introduces a second trade-off. Efficient removal of organic tissue, biofilm, or natural organic matter improves spectroscopic identification, but aggressive chemical treatment can alter or destroy particular polymers. Consequently, recovery validation should encompass the complete workflow—not merely the final instrumental analysis. Hermsen et al. (2018) identified positive recovery controls, negative controls, contamination prevention, treatment validation, and polymer confirmation as core quality criteria for biota analysis. In their evaluation of previously published ingestion studies, mean methodological completeness was only 8.0 of 20 points, while the mean score for reliability criteria was effectively zero, illustrating how incomplete quality assurance and quality control (QA/QC) can constrain the interpretation of otherwise plausible occurrence data.

Particle losses during filtration and sample transfer become increasingly important toward the lower size boundary. In the 2025 international interlaboratory comparison, Ciornii et al. (2025) identified filtration as an unresolved source of uncertainty because small particles could be lost differently among laboratories using different filters and handling procedures. Recovery tests should therefore include representative polymers, morphologies, and—critically—the smallest targeted size fraction rather than validating a method solely with easily recovered large spheres.

Contamination creates error in the opposite direction. Airborne textile fibers, plastic laboratory equipment, polymer-containing filters, clothing, reagents, and sample containers can add particles during processing. The EU drinking-water methodology consequently requires procedural blanks and filtration of liquids used during sample preparation to ≤ 0.45 μm, while recommending direct filtration at the sampling point where practical to minimize handling contamination (European Commission, 2024). Blank correction should nevertheless be reported transparently rather than used to conceal high background contamination; large blank burdens indicate that the analytical workflow itself requires improvement.

### 6.3 Spectroscopic and thermal methods quantify different dimensions of contamination

Spatial resolution differs substantially even among particle-based methods. The international comparison of Ciornii et al. (2025) placed typical lower polymer-identification limits at approximately 10–20 μm for μFTIR and 0.5–5 μm for μRaman, depending on instrumental configuration. Raman therefore extends measurement toward smaller particles, but fluorescence from pigments, biofilms, organic matter, or weathered surfaces can obscure spectra and substantially increase acquisition time. FTIR is generally less affected by fluorescence and is well-established for automated chemical imaging, but its larger diffraction-limited measurement scale means that the smallest MPs accessible to Raman may remain outside the FTIR measurement window.

Quantum-cascade-laser-based infrared imaging, commonly implemented as laser direct infrared (LDIR) analysis, increases analytical throughput by locating particles before acquiring identifying spectra. Routine LDIR particle analysis generally uses a lower size threshold of approximately 20 μm. Ourgaud et al. (2022) reported 80%−100% recovery for reference polyethylene, polypropylene, polystyrene, polyvinyl chloride, and PET particles between 200 and 500 μm after optimization of a marine MP workflow, demonstrating strong performance within the validated size range. This recovery value should not, however, be interpreted as evidence of equivalent performance at 20 μm; instrument detection capability and complete workflow recovery are different analytical characteristics.

Thermal methods address a different question: how much polymer mass is present? Fischer and Scholz-Böttcher (2017) demonstrated simultaneous identification and quantitative determination of common polymer types using Py-GC/MS based on polymer-specific pyrolysis products. Because thermal methods do not depend on optical visualization of an intact particle, they have no meaningful particle-size detection limit in the same sense as μFTIR or Raman. Instead, detection is governed by polymer-specific mass limits of detection (LODs), matrix interference, calibration, and the amount introduced into the analytical system. This difference can be substantial among polymers. In a harmonized Danube monitoring study using thermal extraction–desorption-GC/MS, Kittner et al. (2022) reported LODs of approximately 3.75–6.72 μg for polyethylene, depending on matrix organic content, compared with approximately 0.21–0.23 μg for polypropylene, 0.12–0.16 μg for polystyrene, and 0.07–0.09 μg for poly(methyl methacrylate). Thus, “below detection” for one polymer does not represent the same mass threshold as “below detection” for another. Polymer-specific response factors and interference from environmental compounds must be incorporated before thermal mass concentrations are interpreted comparatively.

Optical microscopy and SEM provide valuable morphological information but should not be treated as equivalent to chemical polymer identification. Visual classification becomes increasingly unreliable as particle size decreases and as environmental aging makes plastics resemble mineral or biological material. SEM can resolve structures at far smaller dimensions than conventional vibrational spectroscopy, but morphology alone does not establish polymer identity; even in the Ciornii et al. (2025) interlaboratory exercise, particles < 10 μm were difficult to assign reliably in SEM images because of poor contrast, overlapping boundaries, and agglomeration. Elemental information from SEM–EDS can help distinguish mineral particles or identify fillers, but it does not provide the molecular specificity needed for unequivocal identification of most organic polymers. The principal advantages and limitations of commonly used MP methods are compared in Table 4.

**Table 4:** Comparative performance, advantages, limitations, and analytical detection boundaries of major microplastic identification and quantification methods.

Technique	Analytical boundary	Major advantages	Principal limitations/uncertainty	Appropriate use	References
Visual/optical microscopy	No universal chemically reliable LOD; identification confidence decreases markedly for smaller and weathered particles	Rapid, inexpensive, preserves morphology, useful for preliminary screening	High observer bias; natural fibers and mineral particles can be misclassified; polymer identity is not chemically confirmed	Preliminary sorting and morphological characterization of relatively large particles	Hermsen et al., 2018; Elkhatib and Oyanedel-Craver, 2020
μFTIR spectroscopy/imaging	Typical lower chemically reliable identification range ~10–20 μm	Non-destructive; established spectral libraries; automated imaging possible; comparatively less affected by fluorescence than Raman	Diffraction-limited spatial resolution; full-filter imaging can be time consuming; weathering and surface coatings may reduce spectral-match quality	Routine chemically confirmed particle analysis above approximately 10–20 μm	Ciornii et al., 2025
μRaman spectroscopy	Typical lower identification range ~0.5–5 μm	Higher spatial resolution than μFTIR; suitable for very small MPs; can characterize pigments and some additives	Fluorescence interference, laser-induced damage, long acquisition times, and matrix interference can compromise identification	Small-particle analysis where adequate matrix removal and fluorescence control are possible	Ciornii et al., 2025
QCL-IR/LDIR imaging	Routine lower threshold approximately 20 μm; 80%−100% recovery reported for 200–500 μm reference particles in one validated marine workflow	High analytical throughput; automated particle location, sizing, and polymer classification	Particles below routine threshold may be missed; performance depends on reflective substrate, spectral library, particle orientation, and sample preparation	High-throughput particle-resolved environmental monitoring	Ourgaud et al., 2022
Py-GC/MS	Mass-based, polymer- and matrix-specific; no intrinsic particle-size LOD equivalent to spectroscopic methods	High chemical specificity; quantifies polymer mass; can detect polymer material below optical particle-size limits	Destructive; no particle number, size, or morphology; calibration, pyrolysis-marker selection, and matrix interference are critical	Mass-based polymer quantification and complementary chemical confirmation	Fischer and Scholz-Böttcher, 2017; Ciornii et al., 2025
TED-GC/MS	Polymer-dependent mass LOD; one environmental workflow reported approximately 3.75–6.72 μg PE, 0.21–0.23 μg PP, 0.12–0.16 μg PS, and 0.07–0.09 μg PMMA	Larger effective sample capacity than conventional Py-GC/MS; useful for complex environmental matrices and integrative mass measurements	Destructive; provides no particle number or morphology; matrix-derived thermal products can interfere; polymer response factors differ	Integrative mass-based monitoring of environmental samples	Kittner et al., 2022
SEM/SEM–EDS	Nanometre-scale imaging is possible, but imaging resolution is not equivalent to polymer-specific detection limit	Excellent surface-resolution and morphology; useful for observing weathering, cracks, coatings, and inorganic fillers	Polymer chemistry generally cannot be confirmed from morphology alone; labor intensive; agglomeration and overlapping particles complicate size measurements	Complementary morphological characterization rather than standalone polymer identification	Ciornii et al., 2025

EDS, energy-dispersive X-ray spectroscopy; GC/MS, gas chromatography/mass spectrometry; LDIR, laser direct infrared; LOD, limit of detection; MPs, microplastics; PE, polyethylene; PMMA, poly(methyl methacrylate); PP, polypropylene; PS, polystyrene; Py-GC/MS, pyrolysis–gas chromatography/mass spectrometry; QCL-IR, quantum cascade laser infrared; SEM, scanning electron microscopy; SEM–EDS, scanning electron microscopy coupled with energy-dispersive X-ray spectroscopy; TED-GC/MS, thermal extraction–desorption gas chromatography/mass spectrometry; μFTIR, micro-Fourier-transform infrared spectroscopy; μRaman, micro-Raman spectroscopy.

### 6.4 Number concentration and mass concentration describe different exposure dimensions

A major source of conceptual inconsistency arises when studies express MP burden in incompatible units. Particle-resolved techniques typically report particles per volume, mass, tissue weight, or individual organism, whereas thermal methods report polymer mass. These metrics are not interchangeable without assumptions about particle dimensions, shape, density, and polymer identity. This difference becomes increasingly consequential as particle size decreases. Fragmentation of one large particle into many smaller particles may dramatically increase particle number and surface area while leaving total polymer mass almost unchanged. A number-based analysis will therefore emphasize the small-particle fraction, whereas a mass-based method may be dominated by relatively few large particles. Neither metric is inherently superior; each represents a different dimension of exposure. Simple number-to-mass conversion based on an assumed spherical particle can introduce substantial error because environmental MPs comprise fibers, films, fragments, foams, and irregular aggregates. Koelmans et al. (2020) developed probabilistic methods that incorporate realistic size, shape, and density distributions to translate among particle number, volume, and mass concentrations. Their analysis showed that methodological rescaling can reduce artificial variability among environmental datasets and improve alignment between exposure and effect studies. Where conversion is necessary, uncertainty distributions are therefore preferable to single deterministic conversion factors. Reporting should consequently include, wherever technically possible, the measured size range, size distribution, shape categories, polymer composition, and number concentration, accompanied by mass information when an appropriate validated method is available. Cowger et al. (2020) similarly recommended harmonized reporting of sampling design, particle dimensions, polymer identity, analytical thresholds, QA/QC procedures, and concentration units to make MP datasets reproducible and computationally comparable.

## 7 From degradation to safe remediation and valorization

The transition from understanding MP fate to designing remediation requires a stricter definition of what constitutes successful treatment. Particle capture, surface erosion, fragmentation, depolymerization, microbial assimilation, mineralization, and conversion into a new product are mechanistically distinct outcomes. A process that removes 99% of particles from water may simply transfer them to sludge; a biological treatment that produces surface pitting may leave most polymer carbon intact; and a depolymerization process may efficiently recover monomers while transferring additives or sorbed contaminants into process water or residues. Remediation should therefore be evaluated by polymer mass balance, transformation products, contaminant fate, residual ecotoxicity, and final disposition, rather than particle disappearance alone. This evidence hierarchy and its integration with safe conversion pathways are summarized in Figure 4.

**Figure 4:**
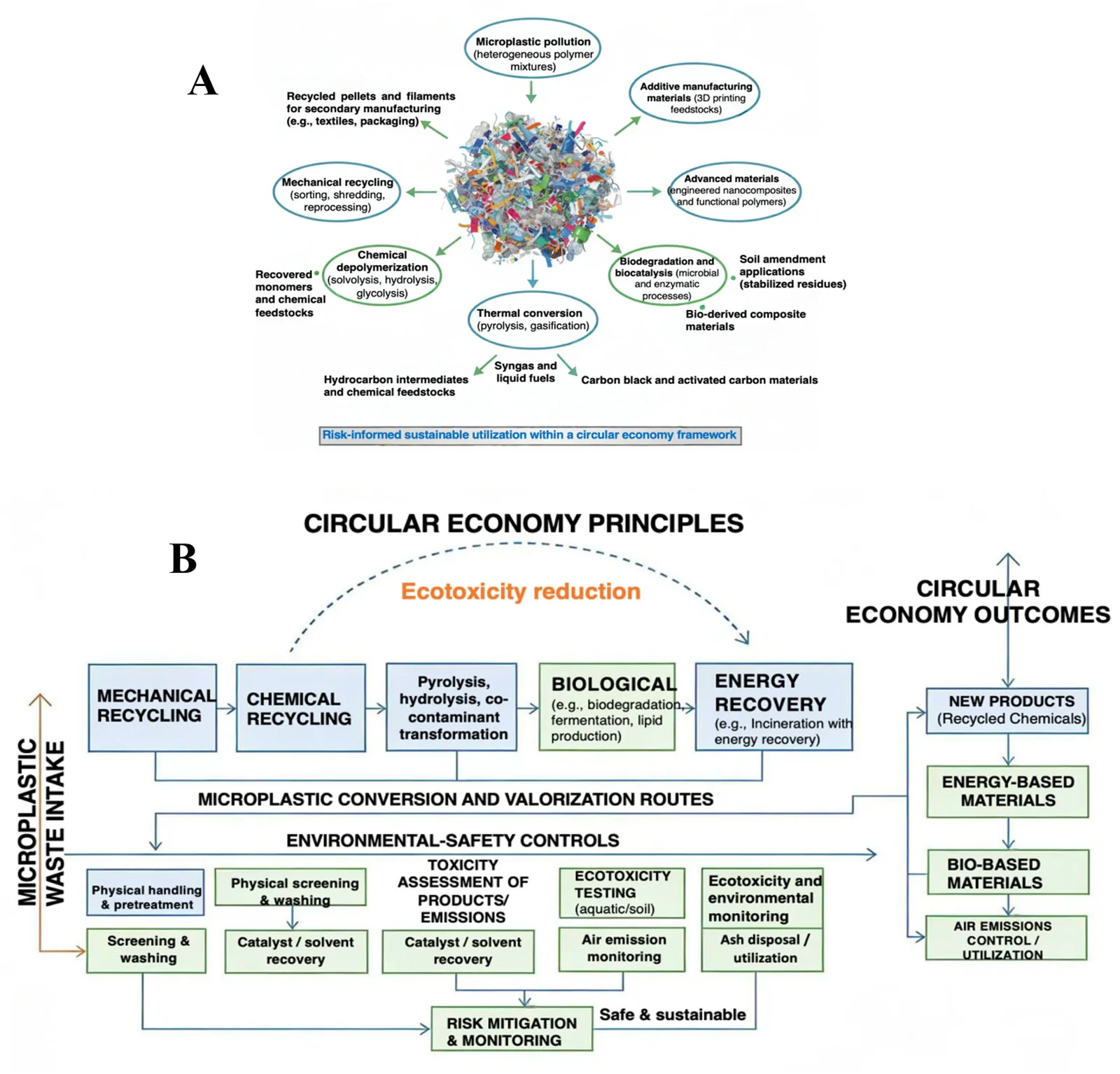
Safe remediation and valorization pathways for microplastic-containing waste streams. **(A)** Major conversion and utilization pathways for heterogeneous microplastic mixtures. **(B)** Risk-informed integration of microplastic conversion and valorization routes within a circular-economy framework.

### 7.1 Degradation must be distinguished from colonization, fragmentation, and mineralization

Microbial colonization is often reported as evidence of plastic degradation, yet Section 4 demonstrated that attachment and biofilm growth can occur without measurable polymer breakdown. Nauendorf et al. (2016) provide a particularly useful counterexample. Polyethylene and nominally biodegradable carrier-bag materials incubated in oxic and anoxic marine sediments for 98 days developed substantial microbial colonization; the biodegradable material supported approximately five-fold greater aerobic and eight-fold greater anaerobic colonization than polyethylene. Nevertheless, neither material exhibited detectable biodegradation based on mass, surface, and chemical analyses. Thus, abundant biofilm development cannot be used as a surrogate for polymer-carbon turnover. Even when biodegradation is unequivocally demonstrated, the rate of mineralization may remain slow. Zumstein et al. (2018) used ^13^C-labeled PBAT to trace polymer-derived carbon directly in agricultural soil. After 6 weeks, approximately 13% of ^13^C-labeled adipate carbon and about 8% of labeled butanediol or terephthalate carbon had been mineralized to ^13^CO_2_. Nanoscale secondary ion mass spectrometry (NanoSIMS) further demonstrated incorporation of polymer-derived ^13^C into microbial biomass. This study provides much stronger evidence for biodegradation than surface deterioration because it documents both microbial assimilation and mineralization, but it also shows that substantial polymer-derived carbon remained after the experimental period. Longer-term experiments reinforce the kinetic limitation. Nelson et al. (2022) followed ^13^C-labeled poly(butylene succinate) in soil for 425 days and measured approximately 65% mineralization of the added polymer carbon. Carbon mass balance and kinetic modeling indicated that hydrolytic cleavage of the polymer, rather than metabolism of released monomers, controlled the overall biodegradation rate. Consequently, even a polyester specifically designed to be soil biodegradable required more than 1 year to reach incomplete mineralization under controlled conditions. The implication for persistent polyolefins such as polyethylene and polypropylene is even more restrictive because their carbon–carbon backbones lack the readily hydrolysable ester bonds present in PBAT or poly(butylene succinate). These results establish a hierarchy of evidence that should be applied consistently to MP degradation studies: colonization, surface modification/biodeterioration, chain scission or molecular-weight reduction, monomer/oligomer release, assimilation of polymer-derived carbon, and mineralization to CO_2_ under aerobic conditions or CO_2_/CH_4_ under relevant anaerobic conditions. Mass loss alone is insufficient because it can reflect additive leaching, detachment of fragments, or dissolution of low-molecular-weight components. Likewise, formation of smaller MPs or nanoplastics represents fragmentation rather than remediation unless the fate of the generated polymer carbon is quantified.

### 7.2 Capture and concentration are prerequisites for treatment but do not constitute destruction

The extreme dilution of MPs in most environmental matrices presents a practical constraint that is frequently overlooked in discussions of valorization. Before enzymatic, microbial, chemical, or thermochemical processing can be considered, dispersed particles must first be recovered and concentrated into a manageable stream. Wastewater treatment provides the clearest demonstration of both the potential and limitation of this approach. Murphy et al. (2016) measured a decline in MP concentration from 15.70 ± 5.23 particles L^−1^ in influent to 0.25 ± 0.04 particles L^−1^ in final effluent, corresponding to 98.41% removal at a secondary wastewater-treatment plant. Despite this high percentage, the remaining effluent was estimated to discharge approximately 65 million MPs per day because of the large water volume processed. Thus, percentage removal alone can substantially overstate environmental protection when the total treated flow is large. Advanced treatment can further reduce particle release. Talvitie et al. (2017) measured 99.9% removal by membrane bioreactor treatment, reducing MP abundance from 6.9 to 0.005 particles L^−1^. Rapid sand filtration achieved approximately 97% removal, dissolved-air flotation approximately 95%, and disc filtration 40%−98.5%, depending on the installation and influent. Importantly, the smallest investigated fraction, 20–100 μm, and fibers remained prominent among particles detected before and after treatment. Capture efficiency therefore depends on treatment technology and particle characteristics rather than operating as a uniform size-independent process. Removal from water must additionally be distinguished from destruction of the polymer. MPs captured during primary clarification, biological treatment, filtration, flotation, or membrane separation are redistributed into sludge, screenings, concentrates, or other residual streams. If these streams are land-applied, inadequately disposed of, or released during subsequent processing, treatment may shift exposure from aquatic to terrestrial systems. A remediation strategy becomes more complete only when the fate of the concentrated polymer fraction is specified. This distinction also changes the logic of valorization. Highly dispersed environmental MPs are unlikely to constitute attractive conversion feedstocks directly because recovery can require processing very large amounts of water, soil, or sediment for relatively little polymer mass. Valorization is therefore most plausible for concentrated MP-rich residuals generated during engineered treatment, industrial processes, filtration systems, or other collection steps, rather than indiscriminate harvesting of low-concentration particles from natural ecosystems.

### 7.3 Enzymatic depolymerization demonstrates what is technically possible—but principally for polyester-rich controlled feeds

Among biological routes, enzymatic polyethylene terephthalate (PET) depolymerization currently provides the strongest quantitative evidence for rapid polymer deconstruction. Tournier et al. (2020) engineered a leaf-branch-compost-cutinase-derived PET hydrolase that achieved at least 90% depolymerization within 10 h, with a terephthalate productivity of 16.7 g L^−1^ h^−1^ using a 200 g PET kg^−1^ suspension and an enzyme loading of 3 mg g^−1^ PET. This represents true depolymerization to recoverable monomers rather than superficial deterioration. Subsequent enzyme engineering extended activity toward less-pretreated materials. Lu et al. (2022) developed functional, active, stable, and tolerant polyethylene terephthalate hydrolase (FAST-PETase), which functioned across approximately 30–50 °C and almost completely depolymerized untreated post-consumer PET from 51 different thermoformed products within 1 week. The recovered monomers were subsequently used to resynthesize PET, providing a closed-loop demonstration. Bell et al. (2022) approached the crystallinity barrier differently by evolving HotPETase to a melting temperature of 82.5 °C, enabling activity near the glass-transition region of PET and more rapid deconstruction of semicrystalline PET than earlier PETases. These advances are significant, but their relevance to environmental MPs requires careful qualification. The feedstocks consisted predominantly of PET films, packaging, thermoformed products, or deliberately prepared materials—not heterogeneous particles recovered from soil, sediment, wastewater sludge, or marine debris. Environmental MPs may contain pigments, fillers, metals, adsorbed chemicals, mineral coatings, biofilms, and polymers at different crystallinities and aging states. Such constituents can interfere with enzyme access, buffer chemistry, downstream separation, microbial metabolism, and product purity. Furthermore, high depolymerization efficiency is not equivalent to mineralization. PET hydrolases convert the polymer principally into terephthalate, ethylene glycol, mono(2-hydroxyethyl) terephthalate, and related soluble products. These products must subsequently be recovered, biologically assimilated, chemically converted, or otherwise managed. From an environmental-remediation perspective, enzymatic hydrolysis therefore represents controlled polymer deconstruction, not complete biodegradation unless the downstream fate of the released carbon is demonstrated.

### 7.4 Biological remediation should target controlled carbon conversion rather than uncontrolled environmental inoculation

The slow rates observed under natural environmental conditions contrast sharply with the much faster kinetics achievable when temperature, enzyme loading, pH, substrate concentration, and mixing are controlled (Bergeson et al., 2024; Bell et al., 2022). This difference argues against viewing environmental release of plastic-degrading microorganisms as a primary remediation strategy (Lensch et al., 2024). *In situ* biodegradation must compete with low substrate availability, nutrient limitation, fluctuating temperature, oxygen gradients, grazing, microbial competition, and restricted enzyme access to hydrophobic or crystalline surfaces (Wang et al., 2024). Controlled treatment systems can instead separate two otherwise confounded functions: extracellular polymer deconstruction and intracellular metabolism of soluble products (Werner et al., 2021). For biodegradable polyesters, isotope-tracing studies demonstrate that microorganisms can incorporate polymer carbon into biomass and mineralize it, but the hydrolytic step may remain rate limiting (Zumstein et al., 2018; Nelson et al., 2022). For PET, engineered hydrolases can accelerate the extracellular step sufficiently that soluble terephthalate and ethylene glycol become suitable substrates for subsequent microbial conversion (Bell et al., 2022). This controlled sequence also improves environmental safety. Enzymes can be immobilized or retained within reactors, while engineered microorganisms can be operated in contained fermentation systems rather than deliberately introduced into open ecosystems (Zurier and Goddard, 2022). Such containment is particularly important for synthetic consortia or genetically modified strains because environmental persistence, horizontal gene transfer, community displacement, and uncontrolled product formation would otherwise introduce new ecological variables into an already complex pollution problem (Mandell et al., 2015; Lensch et al., 2024). A credible biological-remediation endpoint should therefore report at least three independent quantities: fraction of polymer depolymerized, fraction of released carbon assimilated or converted, and fraction mineralized or recovered in a defined product (Bergeson et al., 2024). Without such a carbon balance, high apparent degradation efficiency can conceal accumulation of soluble intermediates or unmeasured smaller fragments (Nelson et al., 2022).

### 7.5 Upcycling begins only after polymer-derived carbon is captured in a higher-value product

Depolymerization becomes upcycling only when polymer-derived carbon is intentionally directed into a product with defined functionality or value. The distinction is important because microbial consumption of a monomer for cell maintenance or complete mineralization to CO_2_ constitutes biodegradation, whereas synthesis of a new chemical or material constitutes valorization. Werner et al. (2021) demonstrated this distinction using engineered *Pseudomonas putida* KT2440. By enabling utilization of ethylene glycol, terephthalate, and bis(2-hydroxyethyl) terephthalate (BHET) and blocking downstream β-ketoadipate catabolism, the engineered strain produced 15.1 g L^−1^ β-ketoadipic acid at a 76% molar yield from commercial BHET. The pathway was subsequently demonstrated with chemically glycolyzed PET, showing that polymer deconstruction and microbial synthesis can be coupled rather than treated as independent operations. A different product route was demonstrated by Sadler and Wallace (2021), who engineered *Escherichia coli* to convert PET-derived terephthalic acid into vanillin. Process optimization increased conversion 157-fold, reaching a maximum vanillin concentration of 119 mg L^−1^ and approximately 79% conversion of terephthalate. Enzymatic PET hydrolysis was then coupled to the pathway using post-consumer bottle material, establishing a direct link between waste PET and a value-added aromatic product. Tiso et al. (2021) extended biological conversion toward new polymer systems by completely hydrolyzing post-consumer PET and feeding the resulting terephthalate and ethylene glycol directly to an adapted *Pseudomonas* strain. The PET-derived carbon was converted into medium-chain-length polyhydroxyalkanoate (mcl-PHA), while engineered production of hydroxyalkanoyloxy alkanoates supplied monomers for synthesis of a partly bio-based poly(amide urethane). This route is conceptually important because it transforms PET into chemically distinct materials rather than merely reproducing PET. Engineered division of labor can further improve simultaneous utilization of mixed monomer streams. Bao et al. (2023) constructed a two-strain *P. putida* consortium in which one population specialized in terephthalate and the other in ethylene-glycol assimilation. Under fed-batch conditions using PET hydrolysate, the consortium produced 637.30 ± 10.14 mg L^−1^ medium-chain-length polyhydroxyalkanoate (mcl-PHA), approximately 92% more than the corresponding single-strain system, while also avoiding ethylene-glycol accumulation. This result demonstrates that microbial ecology can be deliberately engineered to solve metabolic bottlenecks arising during plastic upcycling. However, none of these studies demonstrates direct valorization of heterogeneous environmental MPs. They use commercial intermediates, post-consumer PET, PET powder, purified or crude hydrolysates, or defined reactor feeds. They therefore establish technical feasibility of polymer-derived carbon upcycling, not evidence that environmentally recovered MPs can enter the same process without pretreatment.

### 7.6 Chemical and thermochemical conversion remain relevant for heterogeneous recovered streams

Biological conversion is attractive where the polymer contains enzyme-accessible bonds and where relatively pure monomer streams can be generated. Mixed polyolefin-rich residuals present a different challenge because polyethylene and polypropylene resist hydrolytic depolymerization. For such streams, thermochemical conversion may be more technically appropriate after particles have been concentrated and characterized. Fulgencio-Medrano et al. (2022) studied real plastic-waste streams rather than pristine model polymers. Pretreatment enriched the polyolefin fraction and reduced polyvinyl chloride, PET, metals, inorganic material, and cellulosic contaminants. The most suitable feed produced a 70.6 wt% liquid yield at 460 °C. At 490 °C, the resulting oil had a heating value of 44.3 MJ kg^−1^, a paraffinic content of approximately 65% by GC area, chlorine of 128 ppm, and a diesel-range fraction exceeding 50 wt%. These results demonstrate both the conversion potential of heterogeneous plastic waste and the importance of feed pretreatment: product quality improved when undesirable fractions were removed before pyrolysis.

Catalytic processing can further alter product selectivity. Ratnasari et al. (2017) used staged Mobil Composition of Matter No. 41 (MCM-41)/Zeolite Socony Mobil-5 (ZSM-5) catalysis for high-density polyethylene (HDPE) and obtained an oil yield of 83.15 wt% at a 1:1 catalyst ratio; the oil contained 95.85 wt% aromatics, of which 97.72 wt% fell within the gasoline-range hydrocarbon fraction. Real-world agricultural, construction, packaging, and container plastic wastes were also converted, although their product distributions reflected feed heterogeneity. These results should again be treated as extrapolative evidence for recovered MP-rich streams, because the tested materials were bulk waste plastics rather than environmental MPs. Small particle size may improve heat or mass transfer, but environmental contaminants can introduce opposing constraints: minerals increase ash, metals may alter catalysis, halogenated polymers generate corrosive acid gases, and adsorbed organic contaminants can redistribute into oils, gas, char, or process water. A successful conversion metric must therefore include contaminant partitioning and emissions control, not only polymer conversion or product yield. Chemical or enzymatic depolymerization is preferable when a sufficiently pure polymer stream can be recovered into monomers suitable for closed-loop recycling. Thermochemical approaches may be more tolerant of polyolefin-rich mixtures but require higher energy input and more extensive emissions control. The route should consequently be selected from feed composition and contamination state, rather than from a predetermined preference for biological, chemical, or thermal technology.

### 7.7 Safe valorization requires evidence that hazard is eliminated rather than redistributed

A treatment process cannot be considered environmentally successful if polymer carbon is recovered while associated contaminants are merely transferred into another process stream. Environmental MPs may carry metals, persistent organic pollutants, antibiotics, pigments, flame retardants, plasticizers, mineral matter, biofilms, and other constituents acquired during manufacture or environmental aging. Evidence from real post-consumer plastic waste illustrates the importance of this issue. Kusenberg et al. (2022a) identified nitrogen, oxygen, chlorine, iron, sodium, silicon, and other impurities in pyrolysis oils produced from polyolefin-rich post-consumer packaging; several contaminant concentrations exceeded conventional steam-cracker feed specifications by orders of magnitude. A broader assessment by Kusenberg et al. (2022b) similarly identified nitrogen, oxygen, chlorine, iron, lead, and calcium as major constraints on the direct use of plastic-waste pyrolysis oils because of their potential to promote corrosion, fouling, and catalyst poisoning. Thus, polymer conversion does not imply contaminant elimination, and the fate of associated chemicals must be determined across conversion products and residual streams.

Experimental processing of real plastic waste further demonstrates that pretreatment changes contaminant transfer. The importance of input quality is already established in recycling safety frameworks. The EFSA Panel on Food Contact Materials, Enzymes and Processing Aids (CEP), (2024) for example, requires post-consumer PET recycling assessments to specify characteristics including particle dimensions, non-PET polymer content, dust, moisture, metals, and other input contaminants and to demonstrate decontamination performance through controlled challenge tests. Although those requirements were developed specifically for mechanically recycled PET intended for food-contact applications, the underlying principle is directly relevant to MP valorization: product safety depends on both feed quality and experimentally demonstrated contaminant removal, rather than polymer recovery alone [The EFSA Panel on Food Contact Materials, Enzymes and Processing Aids (CEP), 2024].

Feed composition also determines which treatment route is technically defensible. Kusenberg et al. (2022b) reported that representative plastic-waste pyrolysis oils contained approximately 1,650 ppm nitrogen compared with a typical 100-ppm maximum specification, ~1,460 ppm chlorine compared with 3 ppm, and ~33 ppm iron compared with 0.001 ppm for conventional steam-cracker feeds. Such large differences indicate why dehalogenation, removal of metals and inorganic matter, feed sorting, and subsequent oil upgrading may be necessary before recovered products can re-enter chemical manufacturing. For environmental MPs, which may contain additional weathering products and sorbed contaminants, characterization requirements should therefore be at least as stringent as those applied to conventional post-consumer plastic feedstocks (Kusenberg et al., 2022b). Process performance should then be evaluated using a closed material and hazard balance rather than a single conversion-efficiency value. For chemical or enzymatic depolymerization, the analytical balance should distinguish residual polymer, oligomers, recovered monomers, process-water constituents, and additives or contaminants that remain after conversion. For fermentation-based upcycling, substrate consumption should be reconciled with biomass formation, product carbon, gaseous carbon, and residual metabolites. For pyrolysis and gasification, polymer-derived carbon and relevant contaminants should be tracked among gas, oil, char, aqueous condensates, and gas-cleaning residues. This requirement is particularly important because real plastic-waste pyrolysis oils can contain contaminant concentrations that are incompatible with direct downstream use even when polymer conversion itself is high (Kusenberg et al., 2022a; Fulgencio-Medrano et al., 2022).

Chemical characterization alone may also be insufficient to establish detoxification where the original environmental feed contains complex or incompletely identified mixtures. Post-process ecotoxicity testing can provide an additional safeguard by asking whether the recovered product, aqueous effluent, char, or other residue retains biological activity that would not be apparent from targeted chemical analysis alone. The broader regulatory logic is already established for high-sensitivity recycled products: The EFSA Panel on Food Contact Materials, Enzymes and Processing Aids (CEP), (2024) requires recycling processes intended for food-contact PET to demonstrate decontamination experimentally using surrogate contaminants and to compare residual contamination with conservative migration-based safety criteria. Although environmental-MP valorization involves different exposure scenarios, the same principle applies—decontamination must be demonstrated rather than inferred from polymer conversion efficiency [The EFSA Panel on Food Contact Materials, Enzymes and Processing Aids (CEP), 2024].

Environmental benefit must additionally be demonstrated at the system scale. Uekert et al. (2022) found that the modeled enzymatic PET-recycling system performed approximately 1.2–17 times worse than virgin terephthalic acid (TPA)/PET production across most assessed life-cycle impact categories, with ecotoxicity and fossil-fuel depletion as notable exceptions. Major contributors included post-consumer PET collection and processing, sodium hydroxide consumption, and electricity demand. Importantly, sensitivity analysis showed that higher recovery and process yields, increased solids loading, elimination of energy-intensive pretreatment or pH-control steps, and lower-carbon electricity could reduce impacts toward statistical equivalence with virgin TPA/PET production. Thus, high depolymerization efficiency alone does not establish environmental superiority; energy use, material inputs, recovery efficiency, and downstream processing remain decisive (Uekert et al., 2022). This constraint is likely to be even more important for environmental MPs because the polymer is frequently present at low concentrations in water, sediment, soil, or biological matrices. Capturing and concentrating a dilute contaminant requires energy, water, separation materials, transport, and potentially chemical pretreatment before conversion begins. Accordingly, valorization should not be pursued simply because polymer carbon can technically be recovered. Prevention and source control may provide greater environmental benefit for diffuse emissions, while valorization may be most defensible for concentrated MP-rich residual streams generated by wastewater treatment, industrial filtration, stormwater capture, or other engineered collection systems. This distinction is consistent with life-cycle evidence showing that collection, feed preparation, chemical consumption, and energy demand can materially determine whether a recycling process provides a net environmental advantage (Uekert et al., 2022).

Safe valorization should therefore satisfy three conditions simultaneously: (i) polymer carbon is demonstrably recovered or destroyed; (ii) associated chemical and biological hazards are eliminated, contained, or routed to controlled treatment; and (iii) the overall process provides a net environmental benefit relative to realistic alternatives. Failure of any one condition can produce hazard redistribution rather than remediation. The objective should therefore be risk reduction with resource recovery where justified, rather than resource recovery at any environmental cost. The quantitative evidence distinguishing capture, biodegradation, depolymerization, upcycling, and broader plastic-waste conversion is summarized in Table 5.

**Table 5:** Microplastic-relevant degradation, remediation, and valorization pathways.

Process/endpoint	Feed or experimental system	Evidence	What the result demonstrates	Key limitation for environmental MPs	References
Environmental colonization without degradation	PE and nominally biodegradable bags in marine sediment	Biodegradable material supported ~5 × greater aerobic and 8 × greater anaerobic colonization, but no detectable biodegradation after 98 days	Biofilm formation and high microbial abundance do not establish polymer degradation	Macro-scale bag materials; one marine-sediment setting	Nauendorf et al., 2016
Direct polymer-carbon mineralization	^13^C-labeled PBAT in agricultural soil	Approximately 8%−13% of polymer-derived ^13^C was mineralized to CO_2_ after 6 weeks; polymer-derived C was also incorporated into microbial biomass	Direct evidence of polymer-derived carbon assimilation and mineralization	Biodegradable polyester rather than persistent PE/PP	Zumstein et al., 2018
Long-term biodegradation	^13^C-labeled PBS in soil	Approximately 65% mineralization over 425 days	Even biodegradable polyester may require long timescales; polymer hydrolysis was rate limiting	Controlled soil incubation; polymer designed for biodegradability	Nelson et al., 2022
Conventional wastewater capture	Full-scale secondary WWTP	98.41% removal; concentration decreased from 15.70 ± 5.23 to 0.25 ± 0.04 particles L^−1^, yet approximately 65 million particles day^−1^ were still discharged	High percentage removal does not necessarily mean negligible environmental release	Polymer is transferred into sludge/residual streams rather than destroyed	Murphy et al., 2016
Advanced wastewater capture	MBR, rapid sand filtration, dissolved-air flotation, disc filtration	Approximate removal: 99.9% MBR, 97% sand filtration, 95% DAF, 40%−98.5% disc filtration	Advanced treatment can strongly reduce and concentrate MPs	Capture is not degradation; small particles and fibers may remain	Talvitie et al., 2017
Enzymatic PET depolymerization	Prepared/post-consumer PET	≥90% depolymerization in 10 h; terephthalate productivity 16.7 g L^−1^ h^−1^	Rapid controlled cleavage of PET to recoverable monomers	Optimized PET feed, not heterogeneous environmental MPs	Tournier et al., 2020
Engineered PET hydrolase	51 post-consumer thermoformed PET products	Almost complete depolymerization within approximately 1 week at 30–50 °C	Engineered enzymes can process diverse post-consumer PET and support closed-loop monomer recovery	Environmental aging, mixed polymers, mineral coatings, and sorbed contaminants not represented	Lu et al., 2022
Thermostable PET depolymerization	Semicrystalline PET; engineered HotPETase	Melting temperature increased to 82.5 °C, enabling PET hydrolysis closer to the polymer glass-transition region	Higher enzyme thermostability can improve access to semicrystalline PET	Controlled PET substrate; not demonstrated on environmental MP mixtures	Bell et al., 2022
Biological upcycling to dicarboxylic acid	Commercial BHET/glycolyzed PET → engineered *Pseudomonas putida*	15.1 g L^−1^ β-ketoadipic acid, 76% molar yield from BHET	PET-derived aromatic carbon can be redirected into a higher-value chemical	Requires prior PET deconstruction and controlled fermentation	Werner et al., 2021
Biological upcycling to vanillin	PET-derived terephthalate → engineered *Escherichia coli*	119 mg L^−1^ vanillin, approximately 79% conversion of terephthalate	PET-derived monomer can be converted into a specialty aromatic chemical	Defined substrate and post-consumer PET system; not environmental MPs	Sadler and Wallace, 2021
Biological upcycling to biopolymer	Post-consumer PET hydrolysate → *Pseudomonas*	PET-derived carbon converted into mcl-PHA and a partly bio-based poly(amide urethane)	Demonstrates conversion into chemically distinct polymer products rather than simple PET recycling	Requires hydrolysis and controlled bioconversion	Tiso et al., 2021
Consortium-based biological upcycling	PET hydrolysate → engineered two-strain *P. putida* consortium	637.30 ± 10.14 mg L^−1^ mcl-PHA, approximately 92% higher than the single-strain comparator	Division of labor can improve simultaneous use of terephthalate and ethylene glycol	Defined hydrolysate; engineered contained system required	Bao et al., 2023
Pyrolysis of real mixed plastics	Pretreated polyolefin-rich real plastic waste	70.6 wt% liquid yield at 460 °C; at 490 °C, oil HHV 44.3 MJ kg^−1^, ~65% paraffins, 128 ppm Cl, and >50 wt% diesel-range fraction	Heterogeneous plastic waste can yield useful liquid products when feed is pretreated	Bulk real waste rather than environmental MPs; contaminant carry-over persists	Fulgencio-Medrano et al., 2022
Staged catalytic pyrolysis	HDPE and selected real-world waste plastics	83.15 wt% oil; 95.85 wt% aromatics; 97.72 wt% of oil hydrocarbons in gasoline range under optimized HDPE conditions	Catalytic staging can strongly steer product distribution	Results depend strongly on feed composition and catalyst condition	Ratnasari et al., 2017
Contaminant burden in pyrolysis oil	Post-consumer plastic-waste pyrolysis oils	Representative oils contained about 1,650 ppm N, 1,460 ppm Cl, and 33 ppm Fe, far above typical steam-cracker feed limits	Polymer conversion can retain or concentrate contaminants requiring decontamination/upgrading	Bulk plastic-waste oils; exact contaminant burdens will differ for environmental MPs	Kusenberg et al., 2022a
Feed pretreatment and product quality	Real mixed plastic waste before pyrolysis	Removing PVC, PET, metals, inorganic matter, and cellulose improved polyolefin enrichment and product quality; residual Cl remained measurable	Feed characterization and pretreatment materially affect downstream hazard and product suitability	Pretreatment transfers removed contaminants into other streams that also require management	Fulgencio-Medrano et al., 2022
Life-cycle performance of enzymatic recycling	Modeled enzymatic PET recycling	Base-case environmental impacts were approximately 1.2–17 × higher than virgin TPA/PET production across most assessed categories	High depolymerization efficiency does not guarantee net environmental benefit	Model based on post-consumer PET, not environmental MP recovery	Uekert et al., 2022

^1^^3^C, carbon-13; BHET, bis(2-hydroxyethyl) terephthalate; C, carbon; Cl, chlorine; CO_2_, carbon dioxide; DAF, dissolved-air flotation; Fe, iron; HDPE, high-density polyethylene; HHV, higher heating value; HotPETase, a thermostable variant of *Ideonella sakaiensis* polyethylene terephthalate hydrolase; MBR, membrane bioreactor; mcl-PHA, medium-chain-length polyhydroxyalkanoate; MPs, microplastics; N, nitrogen; PBAT, poly(butylene adipate-co-terephthalate); PBS, poly(butylene succinate); PE, polyethylene; PET, polyethylene terephthalate; PP, polypropylene; ppm, parts per million; PVC, polyvinyl chloride; TPA, terephthalic acid; wt%, weight percent; WWTP, wastewater treatment plant.

## 8 Climate change, circular economy, and sustainability implications

Microplastic pollution and climate change interact through multiple pathways rather than through a single cause–effect relationship. Climate-sensitive variables—including temperature, solar radiation, precipitation, storms, hydrodynamics, drought, and freeze–thaw processes—can alter plastic weathering, fragmentation, transport, biological exposure, and toxicity. Conversely, the plastics life cycle contributes substantially to greenhouse-gas (GHG) emissions, while environmental plastics may influence trace-gas release and marine biogeochemical processes. These interactions mean that MP management cannot be considered independently of climate mitigation, resource efficiency, and circular-economy objectives. At the same time, evidence strength differs markedly among pathways: plastic life-cycle GHG emissions are comparatively well-quantified, whereas climate-driven changes in environmental MP fragmentation and MP-mediated climate feedbacks remain much more uncertain (Zheng and Suh, 2019).

### 8.1 Climate-sensitive weathering and extreme events can alter microplastic generation and redistribution

Temperature and solar radiation interact with oxygen, mechanical stress, water availability, and polymer chemistry to control plastic deterioration. Solar UV radiation initiates photo-oxidation, chain scission, loss of mechanical strength, and ultimately fragmentation into micro- and nanoplastics. Rising temperature can accelerate many chemical reaction rates and alter material properties, while precipitation, freeze–thaw cycles, waves, abrasion, and wind provide the mechanical energy required to fragment embrittled materials. Importantly, however, these processes do not imply a simple relationship in which future warming will automatically produce MPs at a predictable faster rate. Jansen et al. (2024) concluded that quantitative environmental degradation rates remain poorly constrained because UV exposure interacts with temperature, mechanical forces, pollutants, biofouling, and microbial processes. Projected changes in surface UV radiation themselves are generally < 10% over the 21st century under continued Montreal Protocol compliance, further demonstrating that climate–weathering interactions cannot be reduced to UV intensity alone.

Changes in hydrology can produce more immediate effects on MP exposure than gradual polymer degradation. Hitchcock (2020) measured an increase in MP abundance in an Australian estuarine system from approximately 400 to 17,383 particles m^−3^ following heavy rainfall, representing a >40-fold increase. The event mobilized particles accumulated within the catchment and showed that stormwater can produce short-lived exposure pulses that would be missed by steady-state sampling. Similarly, Nakajima et al. (2022) observed an increase in coastal MP abundance from 101,762 ± 13,213 to 23,032,769 ± 8,372,347 pieces km^−2^ 1 day after Typhoon Faxai—approximately 226-fold—while combined micro- and mesoplastic mass increased from 0.070 ± 0.035 to 91.6 ± 30.9 kg km^−2^, an approximately 1,309-fold increase. Concentrations subsequently approached pre-event conditions within several days, demonstrating rapid redistribution rather than permanent local accumulation. These event studies demonstrate that MP distribution is highly sensitive to extreme hydrometeorological forcing, but they should not be presented as direct demonstrations of climate-change causation. A single storm or typhoon cannot be attributed to anthropogenic climate change solely from an MP dataset. Their significance is mechanistic: if climate change modifies the frequency, intensity, seasonality, or geographical distribution of heavy rainfall, flooding, drought–rewetting cycles, storms, and coastal disturbance, the timing and magnitude of MP mobilization are also likely to change. This distinction between event sensitivity and climate attribution is necessary to avoid overstating current evidence (Hitchcock, 2020; Nakajima et al., 2022).

Climate-driven redistribution may also alter where biological exposure occurs. Storm discharge can move MPs from urban and terrestrial reservoirs into rivers and coastal systems, while changing hydrodynamics can resuspend previously deposited particles. Conversely, drought and reduced flows may increase retention in soils, riverbeds, or sediments before subsequent high-flow events remobilize them. Because environmental MPs carry acquired organic matter, contaminants, and microbial biofilms, climate-sensitive redistribution moves not only polymer particles but the particle–chemical–microbial interfaces. The ecological consequence therefore depends on both particle flux and the environmental history of the mobilized material (Jansen et al., 2024).

### 8.2 Warming and other climate stressors can modify MP toxicity

Climate change can also alter MP effects after exposure occurs. Organisms exposed simultaneously to warming, altered light regimes, hypoxia, salinity change, acidification, or nutritional stress may respond differently from organisms tested under standard laboratory conditions. This interaction is important because most MP ecotoxicology has historically tested one stressor at a time. Guilhermino et al. (2021) demonstrated this experimentally during 21-day *Daphnia magna* exposures to 1–5 μm MPs. At the highest MP concentration tested (0.19 mg L^−1^), population growth rate decreased by approximately 27% at 20 °C and 10,830 lx, 38% when light intensity was increased to 26,000 lx, and 59% when temperature was increased from 20 to 25 °C. The estimated EC50 for living offspring decreased from 0.146 mg L^−1^ under the reference temperature/light regime to 0.102 mg L^−1^ under higher light and 0.101 mg L^−1^ under higher temperature. Relative to the baseline experiment, increasing light intensified MP effects by approximately 1.4-fold and increasing temperature by about 2.2-fold. The interaction was not uniformly synergistic, however. Guilhermino et al. (2021) observed antagonism between elevated temperature and MPs at the lowest MP concentration but synergism at the two higher concentrations. This is a critical result because it shows that climate–pollutant interactions cannot simply be represented using a constant “climate amplification factor.” Temperature affects metabolism, feeding, development, oxygen demand, contaminant kinetics, and energy allocation simultaneously, meaning that combined responses may change direction with dose and biological endpoint. Future MP risk assessment should therefore include climate-relevant stressors where there is a plausible interaction with the exposed organism or ecosystem. This does not mean that every toxicity test requires factorial manipulation of all climatic variables. Rather, chronic tests intended to predict future ecological risk should identify the dominant climate-sensitive conditions for the study system—for example temperature and hypoxia in warming aquatic environments or drought–rewetting cycles in soils—and determine whether they alter dose–response relationships. Such designs are more informative than extrapolating MP toxicity measured under a single constant laboratory condition to future ecosystems (Guilhermino et al., 2021).

### 8.3 Plastics contribute to climate forcing, but life-cycle emissions dominate the quantified burden

The climate relationship is bidirectional because plastic production and management themselves generate substantial GHG emissions. Zheng and Suh (2019) estimated global life-cycle emissions from conventional plastics at approximately 1.7 Gt CO_2_-equivalent in 2015. Under continued growth and existing production patterns, their scenario projected emissions reaching approximately 6.5 Gt CO_2_-equivalent year^−1^ by 2050. Their modeling showed that no single intervention was sufficient; coordinated demand management, renewable energy, recycling, and alternative feedstocks were required to prevent rapid growth in the plastics carbon footprint. An independent OECD assessment estimated that fossil-based plastics generated approximately 1.8 Gt CO_2_-equivalent in 2019, equivalent to 3.4% of global GHG emissions, with approximately 90% arising from fossil-fuel-based polymer production and conversion rather than end-of-life treatment (OECD, 2022a). These figures are important for MP management because prevention of MP pollution and climate mitigation share an upstream driver: continued production of virgin fossil-based polymers. Downstream recovery of MPs can reduce environmental leakage, but it cannot by itself offset the GHG burden associated with continually increasing primary-plastic production. Environmental plastic itself can also emit climate-relevant trace gases during weathering. Royer et al. (2018) demonstrated production of methane and ethylene from common plastics exposed to solar radiation. Virgin LDPE incubated for 212 days reached emission rates of approximately 5.8 nmol CH_4_ g^−1^ d^−1^ and 14.5 nmol ethylene g^−1^ d^−1^, and LDPE exposed to air emitted methane and ethylene at rates approximately 2 and 76 times higher, respectively, than material incubated in water. Environmentally aged plastics also produced hydrocarbon gases. These measurements establish a photochemical pathway, but their global climate significance remains uncertain. The rates measured by Royer et al. (2018) are not equivalent to a quantified global emissions inventory, and they should not be presented as comparable in magnitude to the gigatonne-scale emissions associated with plastics manufacture. At present, life-cycle fossil-energy and process emissions are the quantitatively dominant demonstrated climate burden of plastics, whereas direct methane or hydrocarbon emissions from weathering environmental plastics remain incompletely quantified feedback.

### 8.4 Microplastic pollution may interact with climate-sensitive marine biogeochemistry

Further potential feedback operates through microbial and planktonic processes rather than direct GHG release. MPs can be consumed by zooplankton, incorporated into marine snow, colonized by microorganisms, and transported vertically. Because these processes overlap with the biological carbon pump and nutrient cycling, sufficiently widespread MP exposure has the potential to modify marine biogeochemistry. Kvale et al. (2021) explored this possibility using an Earth-system model in which zooplankton ingestion of MPs reduced grazing on phytoplankton. In macronutrient-replete regions, reduced grazing increased phytoplankton export and subsequent remineralization at depth. Their simulations suggested MP-related changes could reduce water-column oxygen inventory by as much as 10% regionally in the North Pacific and contribute an additional 0.2%−0.5% reduction in global ocean oxygen inventory by 2020 relative to 1960, compared with approximately 1% climate-driven oxygen loss simulated over the same period. By 2100, the modeled additional global oxygen loss attributable to MP–zooplankton interactions reached approximately 0.2%−0.7%. These values should be interpreted as hypothesis-generating model results rather than observationally established global effects. Kvale et al. (2021) emphasized large uncertainty in zooplankton selectivity for MPs, environmental MP inventories, aggregation rates, ecosystem structure, and regional nutrient limitation; they explicitly regarded the global biogeochemical implications as qualitative. Their model also did not predict a simple increase in atmospheric CO_2_ drawdown because multiple counteracting nutrient and carbon-cycle effects occurred simultaneously. Nevertheless, the study is important because it illustrates a mechanism through which MP pollution and climate-driven ocean change could interact. Ocean warming and stratification are already altering oxygen inventories and microbial remineralization, while MPs may modify grazing and particle export. Future work should therefore evaluate MP effects not only through organism-level toxicity but also through carbon export, oxygen consumption, microbial-loop activity, and nutrient regeneration, ideally using observations capable of constraining Earth-system models (Kvale et al., 2021).

### 8.5 A circular economy must prioritize prevention and material circulation before environmental recovery

Circularity should not be interpreted simply as developing technologies capable of converting recovered MPs into products. The present global plastics system remains predominantly linear. OECD (2022a) estimated that global plastic production reached 460 Mt in 2019, generating approximately 353 Mt of plastic waste. After accounting for recycling losses, only about 9% of waste was ultimately recycled, while secondary plastics represented only approximately 6% of total plastics feedstock. Thus, valorization of recovered environmental MPs addresses only a very small downstream fraction of a much larger material-flow problem. The highest-leverage circular-economy measures therefore act before plastic becomes diffuse environmental contamination. [United Nations Environment Programme (UNEP), 2023] estimated that a coordinated systems transformation using available approaches could reduce global plastic pollution by approximately 80% by 2040. Within this roadmap, expanded reuse could reduce pollution by about 30%, improved recycling could produce an additional 20% reduction, and reorientation toward alternative delivery systems and carefully selected substitute materials could provide a further 17% reduction. Remaining reductions depend on elimination of problematic and unnecessary uses and improved management of residual and legacy waste. This hierarchy has direct implications for MP management. Preventing tire, textile, paint, pellet, and packaging losses avoids subsequent costs of environmental sampling, capture, decontamination, and conversion. Once MPs are dispersed through oceans, soils, sediments, and the atmosphere, recovering polymer mass becomes progressively more difficult and resource intensive. OECD (2022b) likewise projected that even ambitious policies are much more effective at reducing macroplastic leakage than diffuse MP emissions: under its Global Ambition policy scenario, overall environmental plastic leakage in 2060 was reduced by approximately 85% relative to baseline, whereas modeled MP leakage decreased by only around 9%. This difference highlights why source-specific controls for abrasion and shedding remain necessary even in a substantially more circular material economy. Valorization should consequently occupy a specific position in the hierarchy rather than being treated as the principal circular-economy solution: (i) avoid unnecessary material use, (ii) design for durability and low shedding, (iii) reuse, (iv) recover and recycle concentrated waste, (v) capture unavoidable MP-rich residuals, and (vi) apply controlled valorization where net benefit can be demonstrated. [United Nations Environment Programme (UNEP), 2023] and OECD (2022a, b) both emphasize that recycling is important but cannot compensate for unconstrained growth in virgin-material demand.

### 8.6 Circularity and low-carbon performance are related but not equivalent

A process can recover polymer carbon without necessarily lowering total environmental impact. Uekert et al. (2022) found that their base-case enzymatic recycling system produced 1.2–17 times greater impacts than virgin TPA/PET production across most modeled categories, although improvements in solids loading, recovery efficiency, pretreatment, and renewable electricity substantially reduced those burdens. Circularity therefore describes material retention within the economy; it does not by itself demonstrate climate or environmental superiority. The same trade-off applies to thermochemical recycling. Jeswani et al. (2021) reported that chemical recycling of mixed plastic waste through pyrolysis had approximately 50% lower climate-change impact than energy recovery through incineration. When recyclate quality was considered, its global-warming impact was broadly comparable with mechanical recycling, and production of chemically recycled plastic generated a lower climate burden than an equivalent virgin fossil-plastic route. However, pyrolysis performed worse for several non-climate impact categories. The preferred process therefore depends on the full impact profile rather than carbon emissions alone. Material substitution presents similar complexity. Bio-based plastics can reduce dependence on fossil carbon and, in some systems, lower life-cycle GHG emissions. However, agricultural land demand, fertilizer inputs, biodiversity effects, competition with food production, and unclear end-of-life infrastructure can offset part of that advantage (Rosenboom et al., 2022). Likewise, a material described as biodegradable is sustainable only if degradation occurs under conditions that correspond to its likely end-of-life environment. Law and Narayan (2022) therefore argued that universal or marine biodegradability should not substitute for an explicit recovery and end-of-life strategy. The sustainability criterion for MP management should therefore combine at least four dimensions: reduction of environmental leakage and toxicity, conservation of polymer/material value, net life-cycle GHG performance, and avoidance of burden shifting across other environmental impact categories. This is particularly important for dilute environmental MPs, for which collection and concentration can dominate energy and material demand before valorization even begins (Jeswani et al., 2021; Uekert et al., 2022).

### 8.7 Implications for the Sustainable Development Goals

MP mitigation intersects most directly with several Sustainable Development Goals rather than belonging to a single pollution target. SDG 6 (Clean Water and Sanitation) is supported by reducing MP inputs to freshwater and improving wastewater and drinking-water treatment. SDG 12 (Responsible Consumption and Production) is central because reducing virgin material demand, preventing losses, designing safer and more recyclable products, extending product lifetimes, and improving material recirculation address the upstream drivers of MP formation. UNEP identifies sustainable consumption and production as a central response to the linked crises of resource depletion, climate change, biodiversity loss, and pollution [United Nations Environment Programme (UNEP), 2023].

The climate link connects MP management to SDG 13 (Climate Action). The plastics life cycle already accounts for a measurable fraction of global GHG emissions, meaning that reductions in virgin fossil-plastic demand, increased material efficiency, renewable energy, and genuinely low-impact recycling can deliver both pollution and climate benefits (Zheng and Suh, 2019; OECD, 2022a). However, energy-intensive environmental recovery or conversion that produces greater life-cycle impacts than the displaced alternative would weaken rather than strengthen this synergy. Environmental leakage connects directly to SDG 14 (Life Below Water) and SDG 15 (Life on Land) because MPs alter aquatic and terrestrial exposure, microbial communities, contaminant transport, and ecological processes. The climate-sensitive redistribution described above further links these goals: storm-driven transfer can move land-based pollution into aquatic systems, while warming can change toxicity and microbial transformations. Jansen et al. (2024) explicitly placed plastic pollution and its interactions with UV radiation and climate change within this multi-SDG framework.

Human exposure and contaminant management additionally relate to SDG 3 (Good Health and Wellbeing), but the causal limitations discussed in Section 5 should be retained. Sustainability arguments should not claim quantified human-health benefits from MP removal where disease causality is unresolved. A more defensible interpretation is that preventing environmental exposure and hazardous chemical circulation is consistent with precautionary protection of human health while exposure–response relationships continue to be resolved.

### 8.8 Climate resilience and circularity must be evaluated together

The interaction between climate change and MP pollution is best understood as a set of coupled feedbacks with different levels of evidence. Strong observational evidence demonstrates that rainfall and storms can produce large, transient changes in MP distribution; controlled experiments show that warming can modify biological effects; and life-cycle analyses establish that plastics production contributes materially to global GHG emissions. By contrast, future climate effects on environmental plastic fragmentation and global MP-driven biogeochemical feedbacks remain quantitatively uncertain and should be presented as emerging rather than established climate mechanisms. Circular-economy strategies can address both climate and pollution pressures, but only when they reduce virgin-material demand and prevent leakage rather than simply moving polymer carbon between processes. Recycling and valorization are valuable components of this transition, particularly for concentrated recoverable streams, but they sit downstream of source reduction, product redesign, durability, reuse, and shedding prevention. This ordering is particularly important for MPs because once material has fragmented and dispersed through the environment, recovery becomes increasingly difficult and resource intensive [OECD, 2022a,b; United Nations Environment Programme (UNEP), 2023]. Sustainability claims must therefore be tested against realistic counterfactuals. A valorization process should not be labeled circular merely because it produces a marketable product, nor climate beneficial solely because it avoids incineration. The correct comparison includes the displaced virgin material, collection and concentration requirements, energy mix, chemical inputs, product yield and quality, contaminant fate, residual waste, and non-climate environmental effects. The contrasting LCAs of pyrolysis and enzymatic PET recycling demonstrate how strongly conclusions can depend on these system boundaries (Jeswani et al., 2021; Uekert et al., 2022). For MP management, the resulting sustainability principle is therefore prevention before dispersion, recovery before uncontrolled transformation, and valorization only where net risk and life-cycle burdens are demonstrably reduced. This principle connects the exposure–transformation–risk–management continuum developed throughout this review to climate resilience, circular resource use, and sustainable development.

## 9 Research priorities and decision framework

Future MP research should move beyond documenting occurrence, microbial colonization, and isolated toxicological responses toward resolving the uncertainties that directly influence environmental decision-making. The highest priorities are therefore to improve comparability of exposure data, demonstrate microbial and toxicological functions under environmentally realistic conditions, and establish whether proposed remediation or valorization technologies provide a measurable net reduction in environmental risk.

### 9.1 Standardized and environmentally realistic exposure assessment

A fundamental priority is to improve the comparability of MP measurements across studies. Differences in sampling volume, size cut-off, extraction recovery, contamination control, polymer-identification technique, and reporting units can substantially alter apparent concentrations. The recent interlaboratory comparison of 84 laboratories demonstrated considerable variability even when nominally identical reference materials were analyzed: reproducibility variation reached approximately 45.9%−62% for PET and 62%−117% for polyethylene using thermal approaches, while spectroscopic methods also showed substantial interlaboratory variation (Ciornii et al., 2025). Future studies should therefore report recovery efficiency, procedural blanks, analytical size range, polymer-specific LOD/limits of quantification (LOQs), and measurement uncertainty rather than presenting particle concentration as an exact value. Environmental realism is equally important. Toxicological and microbial studies still rely heavily on pristine spherical particles that differ markedly from weathered environmental MPs in morphology, oxidation, surface roughness, eco-corona formation, biofilm development, and contaminant loading. Beggel et al. (2025) emphasized that harmonized ecotoxicological assessment requires realistic particle diversity, appropriate exposure pathways, comparable dose metrics, and suitable reference particles. Future work should therefore compare field-derived or deliberately aged particles with matched pristine-polymer controls and, where possible, natural-particle controls. This alignment has direct consequences for risk assessment. Coffin et al. (2026) showed that uncertainty associated with matching experimental particles to environmentally relevant particle characteristics can shift estimated ecological hazard thresholds by up to approximately two orders of magnitude. Thus, improved characterization of particle size, shape, polymer, aging state, and environmental context can materially change the numerical interpretation of risk rather than simply improve descriptive completeness.

### 9.2 From microbial presence to demonstrated function and environmentally relevant effects

A second priority is to distinguish microbial presence and genetic potential from measured environmental function. Plastisphere research has established that MPs can support microbial communities that differ from surrounding water, sediment, or soil, but taxonomic enrichment alone does not demonstrate ecological consequence. Genome-resolved metagenomic analysis by Lips et al. (2026), for example, identified conserved plastisphere functional potential across the North Atlantic and North Pacific, including genes associated with carbon utilization, nitrogen metabolism, UV protection, and alternative energy pathways. Such results provide strong evidence of functional potential, but they should be complemented by metatranscriptomics, proteomics, metabolomics, stable-isotope tracing, and direct process-rate measurements before specific environmental functions are inferred. The same distinction is particularly important for antimicrobial resistance. Zhou et al. (2024) moved beyond ARG detection by demonstrating that conjugative transfer in estuarine MP biofilms was 7.2–19.6 times higher than among suspended bacteria. Future studies should apply similar direct-transfer approaches under realistic mixed communities and evaluate how aging, antibiotics, metals, nutrients, salinity, and environmental stress affect ARG persistence and transfer. The key question is not whether resistance genes occur on MPs, but whether plastics increase their transfer or persistence relative to relevant natural surfaces. Biodegradation research requires equally strict evidence. Colonization, surface pitting, FTIR changes, mass loss, or fragmentation should not be interpreted automatically as polymer biodegradation. Direct carbon-tracing studies provide a more rigorous standard. Recently, Bajo et al. (2026) used ^13^C-labeled PE to demonstrate direct mineralization by *Bacillus velezensis* MT9, but the maximum measured ^13^C mass loss was only 0.199% after 14 days under the most favorable tested condition. These studies illustrate why future degradation experiments should quantify polymer-derived carbon in biomass, soluble products, CO_2_ or CH_4_, rather than relying on visual or gravimetric evidence alone. Ecotoxicology should follow the same principle of increasing evidence strength. Molecular biomarkers such as oxidative stress or altered gene expression are mechanistically valuable, but environmental relevance is stronger when these responses are linked to chronic effects on growth, reproduction, behavior, or population performance. Experiments should increasingly use environmentally transformed particles and climate-relevant co-stressors. Guilhermino et al. (2021), for example, found that the effect of MPs on *Daphnia magna* population growth became substantially stronger at elevated temperature, demonstrating that future ecological risk cannot always be inferred from single-stressor tests performed under fixed laboratory conditions.

### 9.3 Decision criteria for remediation, valorization, and adaptive management

Future remediation studies should focus less on percentage removal and more on final particle and hazard fate. A treatment that transfers MPs from wastewater into sludge or from water into a concentrated filter residue has reduced immediate exposure but has not destroyed the polymer. Studies should therefore report full mass balances covering treated water, sludge, concentrates, residuals, generated smaller particles, and associated contaminants. Biological treatment additionally requires carbon balances that distinguish remaining polymer, soluble intermediates, microbial biomass, mineralized carbon, and recovered products. Valorization requires an additional level of evidence. Results from clean polymers, post-consumer plastics, or defined PET hydrolysates should not be presented as direct demonstrations of environmental-MP valorization. Instead, studies should state whether evidence derives from environmentally recovered MPs, deliberately aged model particles, post-consumer waste, or bulk polymer feedstocks. This distinction is essential because environmental MPs are heterogeneous, biofouled, contaminated, and difficult to recover at high purity. For any candidate conversion route, feed characterization should include polymer composition, aging, mineral or ash content, halogens, additives, metals, organic contaminants, and microbial loading. Product yield should then be evaluated together with contaminant fate, emissions, residual ecotoxicity, and product quality. Kusenberg et al. (2022a,b) demonstrated that pyrolysis oils derived from post-consumer plastic waste can contain nitrogen, chlorine, iron, and other contaminants at concentrations incompatible with direct downstream use, showing that high polymer conversion does not guarantee safe product recovery. Life-cycle performance should also become a required decision criterion rather than an optional addition. Uekert et al. (2022) showed that a technically successful enzymatic PET-recycling system performed approximately 1.2–17 times worse than virgin TPA/PET production across most modeled environmental-impact categories in its base case, although process optimization substantially reduced these burdens. This finding illustrates why technological feasibility, circularity, and environmental benefit should be evaluated separately. The resulting management logic is therefore straightforward. Prevention and source control should be prioritized before environmental dispersion; monitoring should establish whether exposure is analytically reliable and biologically relevant; remediation should be selected only when it reduces net exposure without uncontrolled transfer; and valorization should proceed only when polymer recovery, contaminant control, product safety, and life-cycle benefit are demonstrated together. Climate-sensitive monitoring should additionally incorporate event-based sampling because storms and extreme hydrological events can generate large, transient MP exposure pulses that conventional periodic monitoring may miss. In this framework, microbiology contributes far more than polymer degradation alone. Microorganisms influence biofilm formation, contaminant transformation, antimicrobial-resistance exchange, nutrient cycling, polymer-carbon conversion, and engineered upcycling. The most important future step is therefore to connect microbial identity → expressed function → measured process rate → environmental consequence → management decision. This evidence chain provides a more useful basis for future MP research than simply expanding the number of occurrence, sequencing, or toxicity studies.

## 10 Conclusion

Microplastics are not static polymer fragments but environmentally transformed particle–chemical–microbial interfaces whose mobility, reactivity, and biological effects depend on aging, contaminant acquisition, organic conditioning, and microbial colonization. Plastisphere communities can modify contaminant fate, nutrient cycling, antimicrobial-resistance exchange, particle transport, and polymer transformation; however, microbial presence should not be equated with function. Stronger evidence requires direct measurements of process rates, horizontal gene transfer, transformation products, polymer-derived carbon assimilation, and mineralization. Likewise, ecotoxicological effects depend on particle properties, exposure conditions, co-contaminants, and environmental stressors, while analytical heterogeneity in sampling, size cut-offs, recovery, polymer identification, and reporting units remains a major barrier to cross-study comparability. Effective management therefore requires a hierarchy of evidence. Particle capture should be distinguished from destruction, fragmentation from degradation, and depolymerization from true mineralization or upcycling. Although enzymatic and microbial conversion technologies show considerable promise, most high-performance studies still rely on defined polymers, post-consumer plastics, or prepared hydrolysates rather than heterogeneous environmental MPs. Safe remediation and valorization consequently require feed characterization, carbon and contaminant mass balances, product and residue safety testing, microbial containment, and life-cycle verification. Future progress should connect microbial identity to expressed function, particle detection to exposure relevance, degradation to verified carbon fate, and technological performance to net environmental risk reduction. This microbiology-informed framework provides a more defensible basis for integrating MP ecology, ecotoxicology, remediation, and circular-resource management.
